# Circulating Exosomal microRNAs as Biomarkers of Colon Cancer

**DOI:** 10.1371/journal.pone.0092921

**Published:** 2014-04-04

**Authors:** Hiroko Ogata-Kawata, Masashi Izumiya, Daisuke Kurioka, Yoshitaka Honma, Yasuhide Yamada, Koh Furuta, Toshiaki Gunji, Hideki Ohta, Hiroyuki Okamoto, Hikaru Sonoda, Masatoshi Watanabe, Hitoshi Nakagama, Jun Yokota, Takashi Kohno, Naoto Tsuchiya

**Affiliations:** 1 Division of Genome Biology, National Cancer Center Research Institute, Tokyo, Japan; 2 Division of Cancer Development System, National Cancer Center Research Institute, Tokyo, Japan; 3 Department of Gastroenterology, The University of Tokyo Hospital, Tokyo, Japan; 4 Laboratory for Medical Engineering, Division of Materials and Chemical Engineering, Graduate School of Engineering, Yokohama National University, Kanagawa, Japan; 5 Gastrointestinal Oncology Division, National Cancer Center Hospital, Tokyo, Japan; 6 Division of Clinical Laboratories, National Cancer Center Hospital, Tokyo, Japan; 7 NTT Medical Center Tokyo, Tokyo, Japan; 8 Shionogi & Co., Ltd., Osaka, Japan; 9 Institute of Predictive and Personalized Medicine of Cancer (IMPPC), Barcelona, Spain; Kanazawa University, Japan

## Abstract

**Purpose:**

Exosomal microRNAs (miRNAs) have been attracting major interest as potential diagnostic biomarkers of cancer. The aim of this study was to characterize the miRNA profiles of serum exosomes and to identify those that are altered in colorectal cancer (CRC). To evaluate their use as diagnostic biomarkers, the relationship between specific exosomal miRNA levels and pathological changes of patients, including disease stage and tumor resection, was examined.

**Experimental Design:**

Microarray analyses of miRNAs in exosome-enriched fractions of serum samples from 88 primary CRC patients and 11 healthy controls were performed. The expression levels of miRNAs in the culture medium of five colon cancer cell lines were also compared with those in the culture medium of a normal colon-derived cell line. The expression profiles of miRNAs that were differentially expressed between CRC and control sample sets were verified using 29 paired samples from post-tumor resection patients. The sensitivities of selected miRNAs as biomarkers of CRC were evaluated and compared with those of known tumor markers (CA19-9 and CEA) using a receiver operating characteristic analysis. The expression levels of selected miRNAs were also validated by quantitative real-time RT-PCR analyses of an independent set of 13 CRC patients.

**Results:**

The serum exosomal levels of seven miRNAs (let-7a, miR-1229, miR-1246, miR-150, miR-21, miR-223, and miR-23a) were significantly higher in primary CRC patients, even those with early stage disease, than in healthy controls, and were significantly down-regulated after surgical resection of tumors. These miRNAs were also secreted at significantly higher levels by colon cancer cell lines than by a normal colon-derived cell line. The high sensitivities of the seven selected exosomal miRNAs were confirmed by a receiver operating characteristic analysis.

**Conclusion:**

Exosomal miRNA signatures appear to mirror pathological changes of CRC patients and several miRNAs are promising biomarkers for non-invasive diagnosis of the disease.

## Introduction

Colorectal cancer (CRC) is one of the major causes of cancer-related deaths worldwide [1]. Systematic methods for diagnosing pathological conditions might contribute to a high detection rate of patients at early stages of CRC, leading to a reduction in mortality rates. Implementation of the fecal occult blood test and flexible sigmoidoscopy as screening methods has reduced CRC mortality [2], [3]. However, these techniques have inherent limitations; the sensitivity of detection of the fecal occult blood test is fairly low and flexible sigmoidoscopy is invasive and uncomfortable for patients. Carbohydrate antigen 19-9 (CA19-9) and carcinoembryonic antigen (CEA) have been widely used as tumor markers for detection of many types of cancer, including colon, liver, pancreatic, and stomach. However, the sensitivity of these markers for the detection of CRC is low, especially in the early stages of the disease [4]. Therefore, there is a need for the development of CRC-specific diagnostic markers for rapid, non-invasive, and highly sensitive screening of patients.

MicroRNAs (miRNAs) are endogenous, small, non-coding RNAs and their tissue-specific expression contributes to the precise control of various biological processes [5], [6]. Dysfunction of miRNAs is found in various cancers and the endogenous expression profiles of miRNAs can be used to classify cancer types [7], [8]. Several miRNAs showing high levels of expression in cancer tissues have been reported as suitable diagnostic or prognostic markers [9]. Recent studies demonstrated that miRNAs are secreted from various cells, including cancer cells, into body fluids such as blood, urine, breast milk, and saliva, *via* exosomes [10]–[12]. Exosomes are small membrane vesicles of approximately 100 nm that embed protein, lipids, mRNAs, and miRNAs, depending on the origin of the secreting cells [13], [14]. Therefore, exosomal miRNAs in body fluids may be useful diagnostic biomarkers for the detection of cancer [10], [15]. However, there is currently a lack of information regarding the relationship between exosomal miRNA profiles in blood and the pathological condition of cancer patients.

Here, we performed microarray-based profiling of exosomal miRNAs in sera from healthy controls (HCs) and primary CRC patients. The miRNA profiles in exosomes from colon cancer cell lines were also examined to identify candidate biomarkers secreted by colon cancer cells. The exosomal miRNA signatures differed between CRC patients and controls. By comparing the miRNA profiles of clinical samples and cell lines, eight miRNAs (let-7a, miR-1224-5p, miR-1229, miR-1246, miR-150, miR-21, miR-223, and miR-23a) that were significantly elevated in serum exosomes from primary CRC patients and were down-regulated after surgery were identified. CRC-associated elevation of seven of these exosomal miRNAs was validated in an independent sample set using quantitative real-time RT-PCR (qRT-PCR). Taken together, the results presented here demonstrate that exosomal miRNA signatures reflect pathological changes in CRC patients and are applicable for the development of diagnostic strategies for detection of primary CRCs.

## Materials and Methods

### Clinical samples

Serum samples from 88 CRC patients (aged 35 to 65 years) with a primary tumor were provided by the National Cancer Center Hospital Biobank, Japan (Tokyo, Japan) from 2003 to 2004. Serum samples were also collected from 29 of the patients after surgical resection of the primary tumor from 2003 to 2004. For validation by qRT-PCR, serum samples from 13 different CRC patients (aged 45 to 70 years) with a primary tumor were provided by the National Cancer Center Hospital Biobank, Japan in 2009. Surgical specimens of primary colon cancer and surrounding non-cancerous regions were obtained from patients treated at Teikyo University Hospital (Tokyo, Japan) [16]. Sera from 19 individuals (aged 35 to 65 years) that underwent a complete physical screening at the NTT Medical Center Tokyo (Japan) in 2011 were used as HC samples. Serum samples were stored at −20°C until use.

### Ethics Statement

Institutional Review Board approvals for use of the samples were obtained at NTT Medical Center, The University of Tokyo, and National Cancer Center. Written informed consent was obtained from all of the patients included in the study.

### Cell lines

All cell lines used in this study were obtained from the American Tissue Culture Collection. Normal human fetal colon-derived FHC cells were cultured in DMEM-F12 containing 25 mM HEPES, 10 ng/ml cholera toxin, 5 μg/ml insulin, 5 μg/ml transferrin, 100 ng/ml hydrocortisone, and 10% fetal bovine serum [17]. The HCT116, HT-29, RKO, SW48, and SW480 human colon cancer cell lines were cultured in DMEM supplemented with 10% fetal bovine serum [18]–[20]. The culture medium was collected after growth of the cells for 48 h.

### Preparation of exosome-enriched fractions

Exosome fractions were prepared by a step-wise centrifugation-ultracentrifugation method, as described previously with minor modifications [13]. Briefly, culture medium from colon cancer cell lines was centrifuged at 500×g for 5 min to remove the cell debris, and then centrifuged at 16,500×g for 20 min using a JA-30.50 rotor (Beckmann). The cleared supernatant was passed through a 0.20 μm filter and then ultracentrifuged at 120,000×g for 70 min using a TLA-110 rotor (Beckmann). The pellets were washed with phosphate-buffered saline (PBS) and then resuspended in 250 μl of PBS as exosome-enriched fractions. Serum samples (750 μl) from CRC patients and HCs were diluted eight-fold with PBS and the exosome-enriched fractions were prepared as described above. Enrichment of the exosome marker CD81 in the prepared fractions was confirmed by immunoblot analysis (see Methods S1 and Figure S1).

### Preparation of total exosomal RNA

The exosome fraction prepared from serum was mixed with 750 μl of Trizol-LS reagent (Invitrogen) and the aqueous phase was collected by adding chloroform. After the addition of ethanol to the aqueous phase, total RNA was purified using RNeasy Mini Spin Columns (Qiagen). The RNA sample was dried using a speed-vac centrifugal concentrator (Savant) and then dissolved in 10 μl of nuclease-free water. The concentration of RNA was determined using a NanoDrop spectrophotometer (Thermo Scientific) and the Quanti-iT RiboGreen RNA Assay Kit (Invitrogen). The quality of RNA was analyzed using an Agilent 2100 Bioanalyzer and small RNA chips (Agilent Technologies).

### miRNA microarray analysis

Total RNAs from the exosome fractions were labeled with pCp-Cy3 using the Agilent miRNA labeling reagent, and then hybridized to a human miRNA oligonucleotide microarray (8×15 K, version 3.0; Agilent Technologies), according to the manufacturer's instructions. After hybridization, the array was washed with Gene expression Wash Buffer kit (Agilent Technologies) and scanned using an Agilent DNA microarray scanner. After numerical conversion of the raw data using Feature Extraction Software (version 10.7; Agilent Technologies), the transformed data were analyzed using GeneSpring GX software (version 12.5; Digital Biology). The signal intensities of the spots on the microarray were normalized to the total signal intensity of the array and are shown as percentages. The miRNA microarray data have been deposited in the NCBI Gene Expression Omnibus database with accession code GSE40247.

### qRT-PCR

Exosomal miRNAs were reverse transcribed using MultiScribe reverse transcriptase (Life Technologies) and miRNA-specific primers. The miRNAs were quantified by real-time PCR using TaqMan microRNA kits (Life Technologies) and the 7300 Real-Time PCR System (Applied Biosystems), according to a standard protocol [21]. The comparative cycle threshold (Ct) was used to evaluate the relative detection level of each miRNA in the sample, which was determined using the 2^−ΔΔCT^ method [22]. In qRT-PCR experiments, miR-451 was used as an internal standard because it was detectable in all samples and its normalized intensities were not significantly different between HC and CRC serum exosomes (*P*>0.05).

### Statistical analysis

A two-sided paired Student's *t*-test or Welch's *t*-test was used to determine the statistical significance of differences in microarray signal intensities. Hierarchical clustering analysis of the dataset of all miRNA profiles was performed using Ward's method. The receiver operating characteristic (ROC) curve and area under the curve were analyzed to assess the possibility of using a selected miRNA ratio as a diagnostic marker for CRC. Correlation coefficients of exosomal miRNA profiles between cell lines and of the levels of CA19-9, CEA, and exosomal miRNAs in patient serum as candidate CRC markers were determined by multivariate analysis. These analyses were performed using JMP9 software. The Jonckheere-Terpstra test (SPSS software, version 18) was used to determine the correlation between CRC tumor/node/metastasis (TNM) stages and miRNA levels.

## Results

### Identification of exosomal miRNAs that are elevated in CRC by microarray analysis

**Figure 1:**
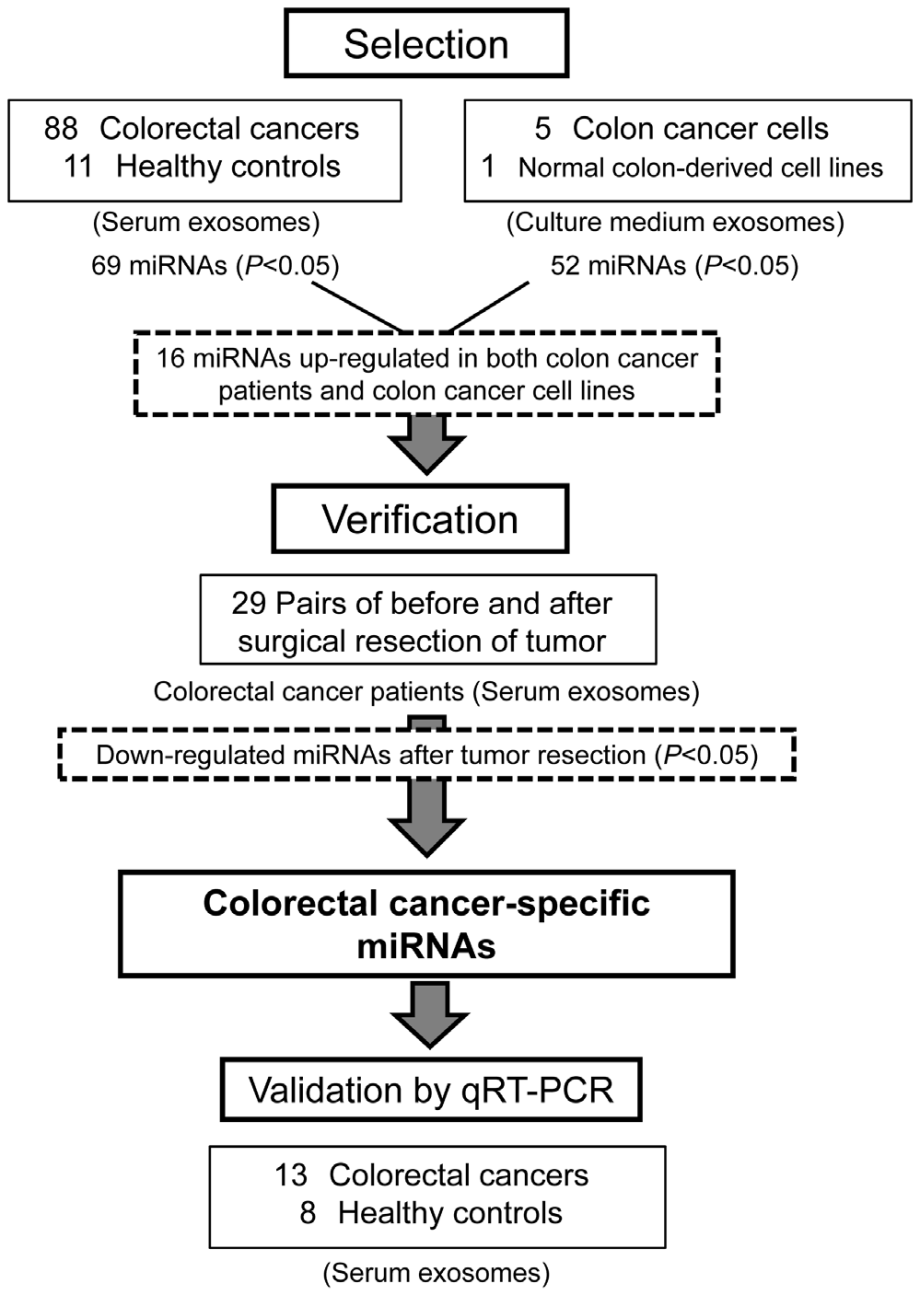
Strategy for the identification of CRC-specific exosomal miRNAs.

Microarray-based screening was used to detect miRNA levels in exosomal fractions of serum samples from CRC and HC patients, as well as culture media from a normal colon-derived cell line and five different human colon cancer cell lines. Figure 1 shows an overview of the screening strategy used. The exosome-enriched fractions were prepared using a step-wise centrifugation-ultracentrifugation method, as shown in Figure S1A. Enrichment of small RNA and CD81 in the exosome fractions was confirmed by capillary electrophoresis and immunoblot analysis, respectively (Figures S1B–D). A multivariate analysis and correlation plots of microarray data indicated reproducible detection of exosomal miRNAs in independent experiments and correlations between the miRNA profiles of the different cancer cell lines (Figure S1E and Table S1). The exosomal miRNA profiles of the five colon cancer cell lines and the FHC cell line were distinct from the endogenous cellular miRNA profiles (Figure S1F).

The miRNA profiles of the exosome fractions of serum samples from 88 primary CRC patients (including TNM clinical stages I, II, IIIa, IIIb, and IV) and 11 HCs were determined using microarrays. The characteristics of the patients and the exosomal miRNAs detected in each group are shown in Table 1. A total of 164 miRNAs were detected in all serum samples examined (Figure S2A) and 69 miRNAs were expressed at significantly higher levels in CRC patients than HCs (*P*<0.05) (Figures S2B and S2C, Table S2). Analysis of the exosomal miRNA profiles of culture media from five different colon cancer cell lines and normal colon epithelial FHC cells revealed that 52 miRNAs were secreted at significantly higher levels from all five colon cancer cell lines than from the FHC cells (Figures S3A and S3B, Table S3).

**Table 1:** Characteristics of the HC and CRC patients included in the study.

	HC	CRC				
Clinical stage		TNM I	II	IIIa	IIIb	IV
	(n = 11)	(n = 20)	(n = 20)	(n = 20)	(n = 16)	(n = 12)
Age in years, mean (SD)	51.0 (9.2)	52.9 (7.8)	57.7 (7.9)	54.6 (7.2)	54.8 (8.6)	56.9 (4.7)
Range	35–64	38–65	35–65	36–63	36–65	48–64
Sex, n (%)						
Female	3 (27.2)	9 (45.0)	7 (35.0)	9 (45.0)	4 (25.0)	4 (33.3)
Male	8 (72.7)	11 (55.0)	13 (65.0)	11 (55.0)	12 (75.0)	8 (66.7)
Number of exosomal miRNAs detected, mean (SD)	62.5 (20.6)	80.9 (18.0)	76.6 (23.7)	74.7 (19.5)	78.3 (18.6)	71.2 (12.1)
Range	61–124	66–111	61–124	66–111	61–124	66–111
Total signal intensities of exosomal miRNAs, mean (SD)	1954 (877)	2037 (784)	1935 (918)	1924 (796)	1878 (854)	1817 (925)

**Figure 2:**
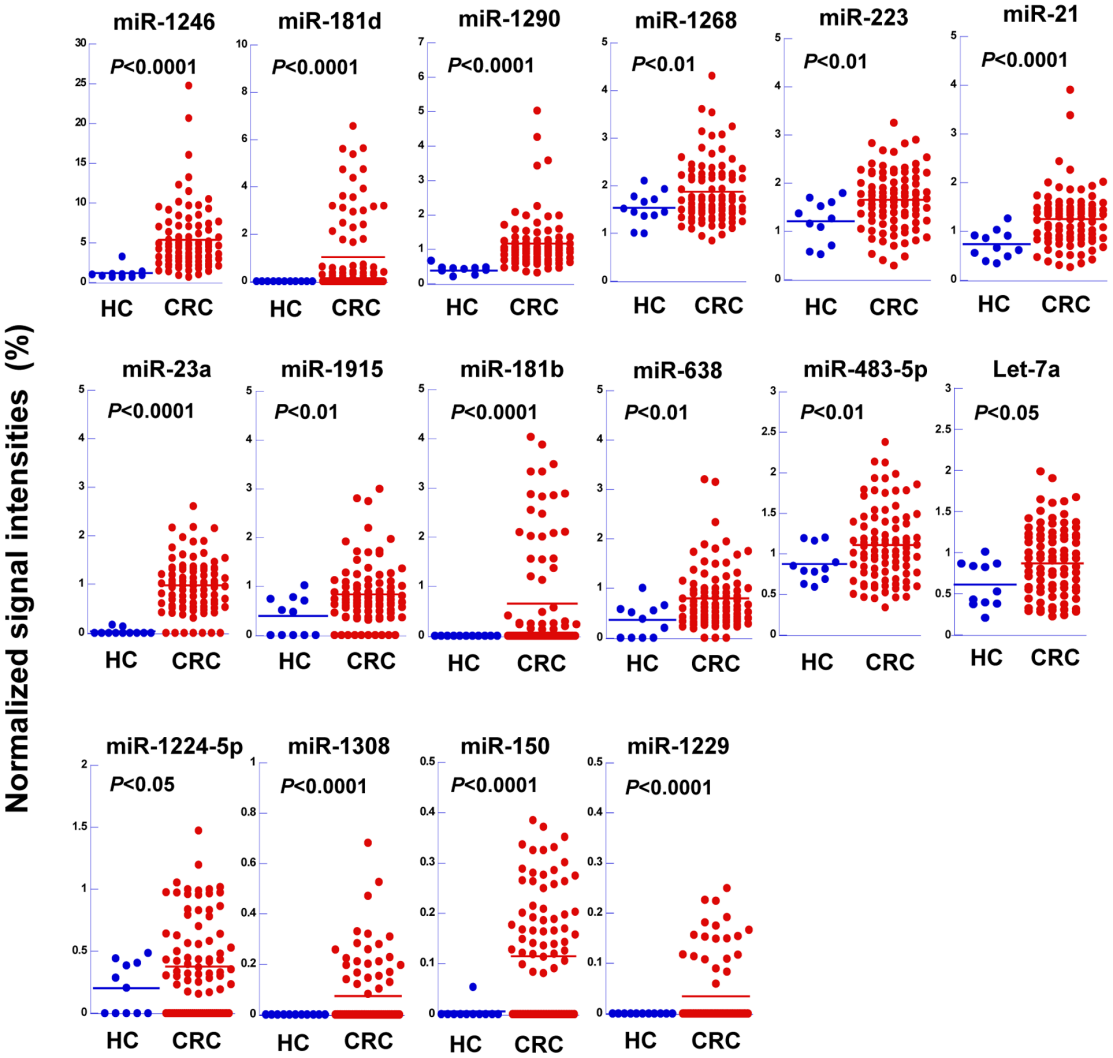
Serum exosomal expression levels of 16 miRNAs that were up-regulated in colon cancer. Serum exosomal miRNA levels in 11 HCs (blue) and 88 CRC patients (red) at different TNM stages (I to IV). The signal intensities were normalized to the total signal intensity of the microarray. The horizontal lines indicate the mean normalized signal intensity for each group. Statistically significant differences were determined by Welch's *t*-test.

A comparison of the 69 up-regulated miRNAs from CRC patients and the 52 up-regulated miRNAs from the colon cancer cell lines revealed that 16 miRNAs were present in both sets (Figure 2): let-7a, miR-1224-5p, miR-1229, miR-1246, miR-1268, miR-1290, miR-1308, miR-150, miR-181b, miR-181d, miR-1915, miR-21, miR-223, miR-23a, miR-483-5p, and miR-638. The endogenous expression levels of these miRNAs were then measured in the FHC and colon cancer cell lines, as well as colon cancer and matched non-cancerous tissues from four additional patients. With the exception of miR-181d, which was significantly up-regulated in both the cancer cells and tissues none of these miRNAs were expressed in the cancer tissues (Figure S3C) or cancer cell lines (*P*>0.05) (Figure S3D) at significantly higher levels than the matched non-cancerous tissues or the FHC cell line, respectively. These results indicate that secretion of miRNAs is not dependent on their cellular expression levels and suggest that up-regulated endogenous miRNAs in cancer cells are not necessarily substrates for exosome-mediated secretion. Colon cancer cells appear to secrete a subset of miRNAs into extracellular spaces via exosomes.

### Relationship between the serum exosomal levels of 16 miRNAs and the clinical stages of colon cancer

Next, we analyzed the relationship between the 16 commonly up-regulated miRNAs and clinical stages of CRC by separating the CRC patients on the basis of their TNM stage. For each miRNA, no significant correlation (*P*>0.05) between the exosomal level and the clinical stage of the disease was identified by a Jonckheere-Terpstra test (Figure S4).

### Down-regulation of eight miRNAs after removal of primary tumors

**Figure 3:**
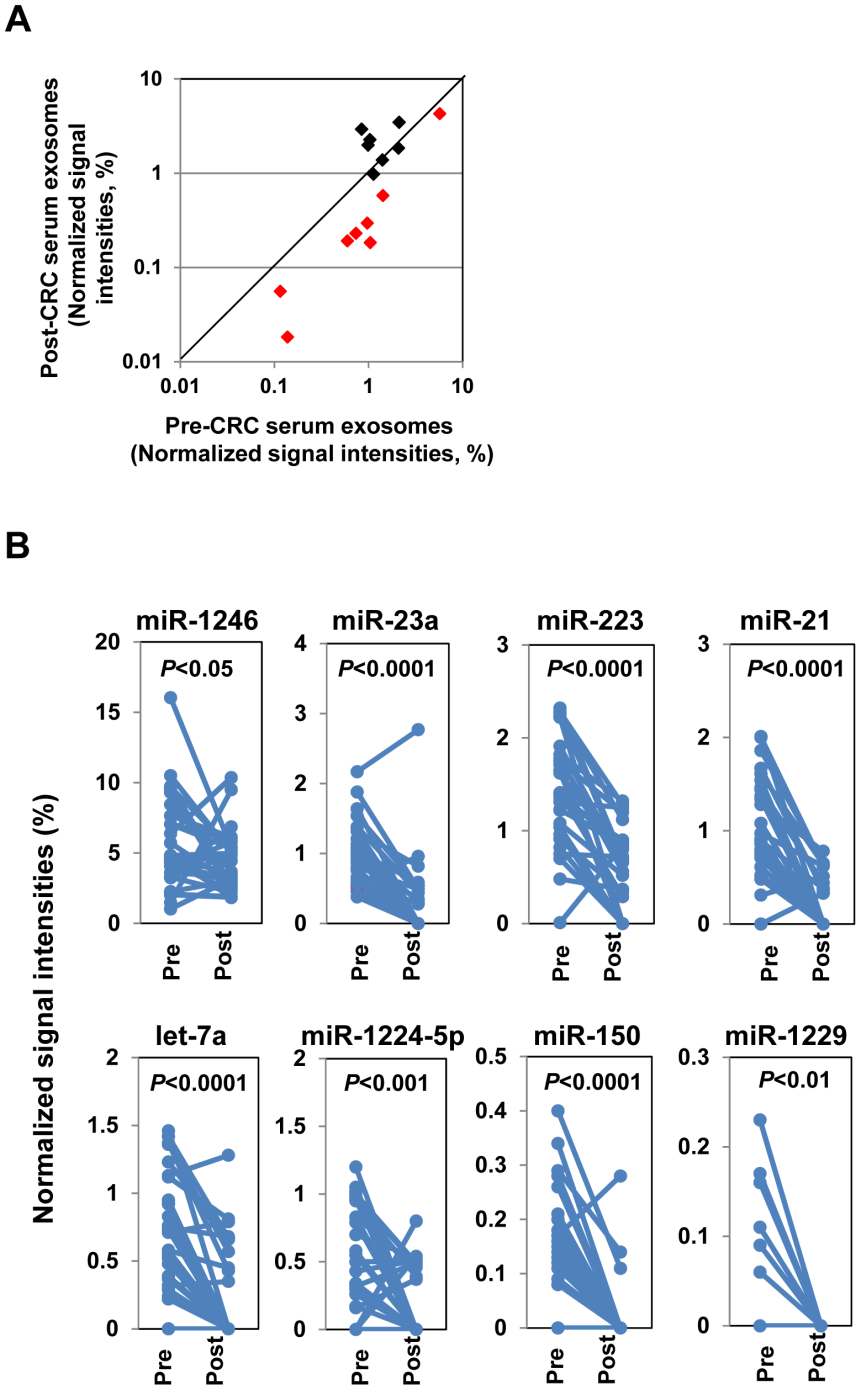
Exosomal miRNA levels in matched serum samples from CRC patients before and after tumor resection. (A) Scatter plot of the 16 commonly up-regulated serum exosomal miRNAs in CRC patients (n = 29) before (Pre) and after (Post) surgical removal of tumors. The sample set included stage I (n = 6), stage II (n = 6), stage IIIa (n = 5), stage IIIb (n = 9), and stage IV (n = 4) patients. The data represent the mean normalized signal intensities (%). The red dots indicate the eight miRNAs that were significantly down-regulated after tumor resection: let-7a, miR-1224-5p, miR-1229, miR-1246, miR-150, miR-21, miR-223, and miR-23a. (B) Individual changes in the serum exosome levels of the eight down-regulated miRNAs in CRC patients (n = 29) before (Pre) and after (Post) surgical removal of tumors. Statistically significant differences between the mean Pre values and the mean Post values were determined by paired Student's *t*-tests.

To determine whether the 16 commonly up-regulated miRNAs originated from cancer cells, microarray analyses were used to determine the levels of these miRNAs in exosome-enriched fractions of matched serum samples collected after surgical resection of primary tumors (n = 29 patients). Expression levels of eight of the miRNAs were significantly reduced (*P*<0.05) after removal of the primary tumors (Figure 3 and Table S4): let-7a, miR-1224-5p, miR-1229, miR-1246, miR-150, miR-21, miR-223, and miR-23a. These results suggest that these eight miRNAs may be derived from exosomes secreted by cancer cells and that their levels may reflect the colon cancer status of patients.

### Potential serum exosome miRNAs for application as diagnostic biomarkers

**Figure 4:**
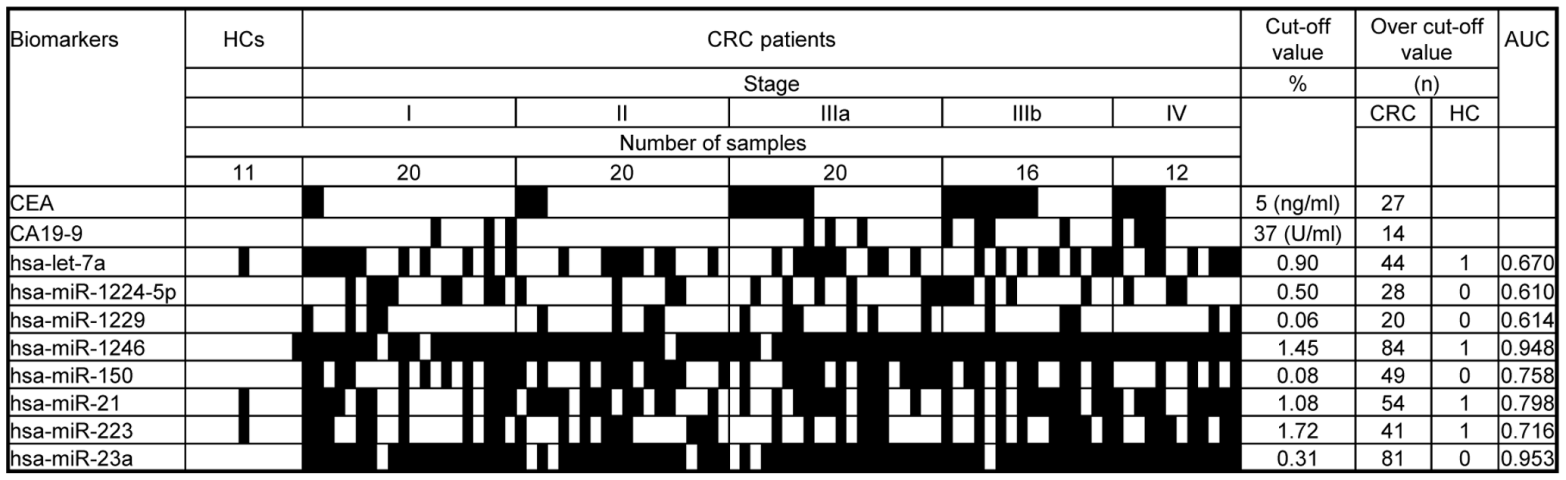
ROC curve analysis of eight miRNAs in serum exosomes of HCs and CRC patients. The signal intensities of the miRNAs are shown as percentages of the total signal intensity. The cut-off values of the eight miRNAs that were up-regulated in colon cancer and down-regulated after tumor resection were analyzed using a ROC curve. Black boxes indicate patients over the cut-off value of the biomarkers or miRNA levels. The normalized intensities of undetectable miRNAs in serum exosomes were calculated as 0.

**Figure 5:**
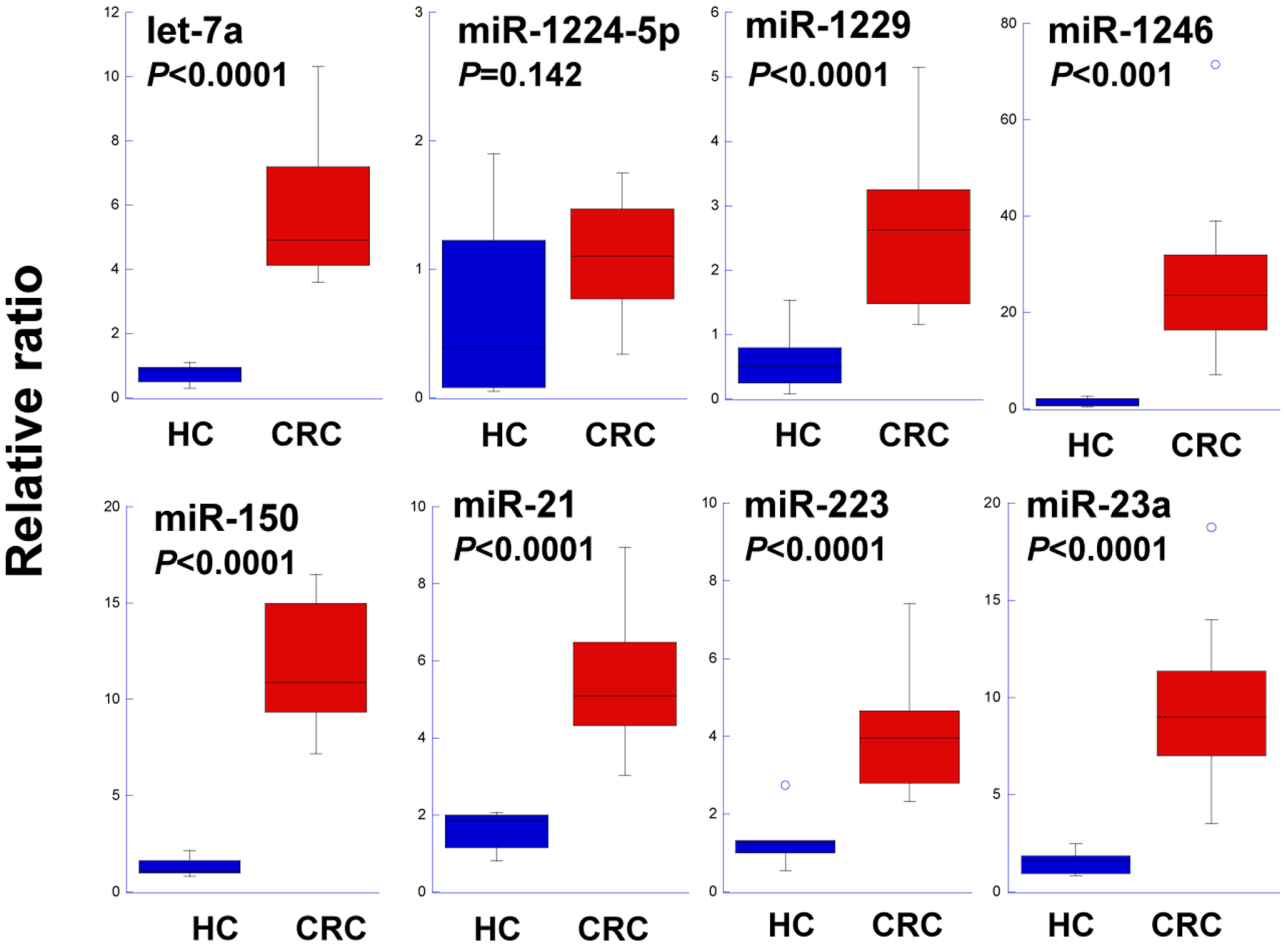
Validation of CRC-associated increases in the expression of eight miRNA in serum exosomes by qRT-PCR. Box-and-whisker plots of the expression levels of the eight selected miRNAs in an independent set of HCs (n = 8) and CRC patients with primary tumor (n = 13). Statistically significant differences between the HC and CRC datasets were determined by Welch's *t*-tests. The comparative cycle threshold (Ct) method was used to quantify the levels of exosomal miRNAs in HC and CRC patients. The relative ratio was calculated using the 2^-ΔΔCt^ method. The Ct value of miR-451 was used as an internal standard. Each data point was normalized to a representative HC sample.

To estimate their power as potential diagnostic markers, the cut-off values of the eight miRNAs that were up-regulated in colon cancer and down-regulated after tumor resection were analyzed using a ROC curve (Figure 4). The true positive rates of miR-1246 and miR-23a for identification of the 88 CRC patients were 95.5% and 92.0%, respectively; these miRNAs also had low false positive rates for identification of the 11 HCs (9% and 0%, respectively). The true positive rates of miR-21, miR-150, let-7a, miR-223, miR-1224-5p, and miR-1229 for identification of CRC were 61.4%, 55.7%, 50.0%, 46.6%, 31.8%, and 22.7%, respectively; the false positive rates of these miRNAs ranged from 0% to 9% (Figure 4). It is noted that a combined usage of 8 miRNAs did not show more diagnostic power than those in miRs-1246 and -23a (data not shown). By comparison, the sensitivities of CEA and CA19-9, which are known biomarkers of CRC, were 30.7% and 16.0%, respectively. A multivariate analysis indicated no correlation between the levels of the eight selected miRNAs in serum exosomes and serum levels of CEA and CA19-9 (Table S5). Therefore, we re-evaluated the expression levels of the eight miRNAs in serum exosomes by qRT-PCR analyses of an additional sample set that included eight HCs, seven stage I CRC patients, and six stage II CRC patients (Table S6). The levels of seven of the miRNAs, namely let-7a, miR-1229, miR-1246, miR-150, miR-21, miR-223, and miR-23a, were significantly higher in serum exosomes from CRC patients than those from HCs (*P*<0.05) (Figure 5). Statistically significant increases in expression levels of these miRNAs were even observed for the early stage (TNM stage I) CRC samples (Figure S5). These results suggest that serum exosomal miRNAs may be useful for the early detection of primary CRCs.

## Discussion

Recently, miRNAs have attracted major interest as a means to analyze the molecular pathways involved in cancer development and progression. In addition to their important cellular functions, it is possible that secreted miRNAs embedded in exosomes may be diagnostic biomarkers for cancer detection. Here, comprehensive analyses of exosomal miRNA profiles identified 16 miRNAs that were expressed at significantly higher levels in colon cancer cell lines and serum samples from CRC patients than normal colon-derived cells and serum samples from HCs, respectively. Endogenous expression levels of most of these miRNAs were not up-regulated in cancer cell lines and cancer tissues, suggesting that exosomal secretion of miRNAs does not depend on cellular expression levels. On the other hand, miR-181, a context-dependent cancer-associated miRNA [23], [24], only indicated up-regulated cellular and exosomal expression levels in cancer cell lines and clinical samples, suggesting an interesting hypothesis that the function of exosomal miR-181 might be involved in colon cancer development and progression.

The serum exosomal levels of eight of these 16 miRNAs were significantly down-regulated after surgical resection of primary tumors, suggesting that these miRNAs are secreted from tumor cells. However, we do not exclude the possibility that these miRNAs are secreted from other cells, including immune and inflammatory cells, which occur coincidentally at primary lesions of the tissue. Finally, CRC-associated elevations of the exosomal levels of seven miRNA levels (let-7a, miR-1229, miR-1246, miR-150, miR-21, miR-223, and miR-23a) were validated by qRT-PCR, indicating that these miRNAs may be suitable biomarkers to detect colon cancers. The high sensitivities and specificities of these exosomal miRNAs determined by ROC analysis (Figure 4) support their use as diagnostic biomarkers.

The exosomal levels of the seven selected miRNAs were not dependent on the clinical stage of CRC (Figure S4). Indeed, miR-23a and miR-1246 showed high sensitivities for stage I samples of 95% and 90%, respectively. By comparison, the sensitivities of CA19-9 and CEA for stage I CRC are only 10 and 15%, respectively. Moreover, qRT-PCR demonstrated reproducible detection of these miRNAs at high levels in an independent set of serum exosomes from stage I CRC patients (Figure S5). These findings suggest that these miRNAs are suitable biomarkers for the detection of early stage CRC. However, further validation is required to support this proposal.

A recent report demonstrated that let-7a is expressed at high levels in exosomes secreted into the serum by glioblastoma tumor cells [15]. In addition, miR-1246 is detectable in whole serum samples, rather than exosome fractions, from esophageal cancer patients [25]. We also measured let-7a, miR-1224-5p, and miR-150 levels in the culture medium of pancreatic cell lines and found that the levels were similar to those secreted by the five colon cancer cell lines (data not shown), suggesting that these miRNAs are commonly secreted from various cancer cells. Several reports have suggested the use of circulating miRNAs in whole plasma or serum, including miR-141, miR-21, miR-221, miR-29, and miR-92a, as diagnostic biomarkers of CRC [26]–[30]. These miRNAs were shown to display sensitivities and specificities comparable to those of the colon cancer markers currently in use. Notably, miR-141, miR-221, and miR-21 were reported to be up-regulated in cancer tissues [31]–[33]; the total miRNA set from whole plasma or serum may include endogenous cellular miRNAs derived from broken or circulating tumor cells [34], [35], which may explain why miR-141, miR-221, and miR-21 are present at high levels in whole serum or plasma samples from cancer patients. In addition, serum/plasma miRNAs, which are not associated with vesicles, were reported to show differential stability to treatment by RNase A [36], suggesting that exosomal miRNAs are more preferable as biological specimens for developing diagnostic biomarkers because of their stability in serum/plasma. In fact, the results presented here demonstrate that exosomal miRNA levels also reflect cancer-bearing status and pathological changes of patients. Exosomes are actively released from cancer cells and their specific constituents depend on the cell from which they originate. Indeed, cancer cells may use an as yet unidentified mechanism to embed a subset of miRNAs into exosomes.

In summary, attempts have recently been made to use miRNAs in serum or plasma as diagnostic biomarkers of various cancers [37]; however, there is currently no collective view of which miRNAs should be selected as markers. The unique properties of exosomes, including their ability to embed specific miRNAs, their stability in circulation, their reproducible detection and, most importantly, the fact that they reflect the properties of cancer cells, may render them useful for the development of highly sensitive diagnostic strategies for rapid and non-invasive monitoring of the pathological condition of cancer patients.

## Supporting Information

Figure S1 Preparation of exosome-enriched fractions from serum or cell culture medium. (A) Overview of the exosome-enrichment procedure. (B) Immunoblot analysis of CD81 levels in human sera prior to exosome-enrichment and before (lane 2) or after overnsight storage at 4°C (lanes 5 and 6), −20°C (lanes 7 and 8), or −80°C (lanes 9 and 10). Fetal bovine serum (FBS) before (lane 1) and after overnight storage at 4°C (lanes 3 and 4) was used as a control. The exosome-enriched fraction was then prepared from 1 ml of serum using the ultracentrifugation method described in the Materials and Methods section (lane 11). Five microliters of each serum sample, or 300 ng of the enriched fraction was loaded onto a 10–20% gradient SDS-polyacrylamide gel. CD81, an exosome marker, was detected using a specific antibody. (C) Detection of the small RNA fraction of cellular total RNAs (endogenous) and exosomal RNAs (exosomal) from HCT116 cells (30 ng each). The RNAs were loaded onto 5–150 nt small RNA chips (Agilent) and capillary electrophoresed using an Agilent 2100 Bioanalyzer. A small RNA fraction containing miRNAs was detectable at approximately 10–40 nt. The peak at 4 nt represents a size marker. The filled and open arrows indicate 5S tRNA and small rRNAs, respectively. FU, fluorescence units. (D) Immunoblot analysis of CD81 expression in serum-free culture medium of HCT116 cells before and after exosome-enrichment. The specified amounts of protein samples from the cell pellet, culture medium, and exosome-enriched fractions were subjected to immunoblot analysis using antibodies targeting CD81 or anti-γ-tubulin, a representative intracellular protein. Lanes 1–4, cell pellet; lanes 5–7, serum-free culture medium; lanes 8–10, exosome-enriched fraction. (E) Scatter plots of normalized signal intensities (%) of exosomal miRNAs (left panels) and endogenous cellular miRNAs (right panels) in the indicated cell lines. (F) Hierarchical clustering of endogenous and exosomal miRNAs in the normal FHC cell line and five human colon cancer cell lines. The data represent the mean values of normalized signal intensities (%) of n = 3 independent experiments. A total of 489 miRNAs were detectable in all samples.
(TIF)

Figure S2 Microarray analysis of miRNA profiles in exosome-enriched serum samples from CRC patients and HCs. (A) Hierarchical clustering of exosomal miRNAs in samples from 11 HCs and 88 CRC patients (TNM: stage I, n = 20; stage II, n = 20; stage IIIa, n = 20; stage IIIb, n = 16; stage IV, n = 12). The blue and red shading indicates the HC and CRC patients, respectively. The signal intensities of each miRNA are shown as a percentage of the total signal intensity on the array. A total of 64 miRNAs were detected in all serum samples examined. (B) Hierarchical clustering of the 69 miRNAs that were expressed at significantly higher levels in CRC patients than HCs (*P*<0.05 by Welch's *t*-test). (C) Scatter plot of the normalized signal intensities of the 69 exosomal miRNAs that were up-regulated in CRC patients (y-axis) compared with HCs (x-axis). The data represent the mean normalized signal intensities of each miRNA from the CRC and HC patients.
(TIF)

Figure S3 Microarray analysis of exosomal miRNAs from colon cancer cell lines and endogenous expression levels of miRNAs. (A, B) The expression levels of the 52 miRNAs that were secreted from five colon cancer cells at significantly higher levels than from FHC cells (*P*<0.05). The data represent the mean normalized signal intensities of n = 3 independent microarray experiments. (C) Scatter plot of the normalized signal intensities (%) representing the endogenous expression levels of the 16 miRNAs that were commonly up-regulated in cancer cell lines and serum samples from CRC patients. The endogenous expression levels of these miRNAs were measured in FHC cells and five colon cancer cell lines The data represent the mean normalized signal intensities of n = 2 independent experiments. (D) Scatter plot of the normalized signal intensities (%) representing the endogenous expression levels of the 16 miRNAs that were commonly up-regulated in cancer cell lines and serum samples from CRC patients. The endogenous expression levels of these miRNAs were measured in cancerous lesions (tumor tissues) and matched normal tissue sections from CRC patients. The data represent the mean normalized signal intensities of each miRNA in four different CRC patients.
(TIF)

Figure S4 The relationship between the TNM stage and expression levels of the 16 up-regulated miRNAs. Serum exosomal miRNA levels in 11 HCs and 88 CRC patients, classified according to the TNM stage of the disease (stage I, n = 20; stage II, n = 20; stage IIIa, n = 20; stage IIIb, n = 16; and stage IV, n = 12). The signal intensities were normalized to the total signal intensity of the microarray. The horizontal lines indicate the mean normalized signal intensity for each group.
(TIF)

Figure S5 Validation of CRC-associated increases in the expression of seven miRNAs in serum exosomes by qRT-PCR. Expression levels of seven selected miRNAs were validated in an independent set of serum exosomes from HCs (n = 8) and TNM stage I (n = 7) and stage III (n = 6) CRC patients. The comparative Ct method was used to quantify the levels of exosomal miRNAs in HC and CRC patients. The relative ratio was calculated using the 2^−ΔΔCt^ method. The Ct value of miR-451 was used as an internal standard. Each data point was normalized to a representative HC sample. Statistically significant differences were determined by Welch's *t*-test. Comparisons between the HC and stage III data were also made and all differences were statistically significant (*P*<0.05)
(TIF)

Method S1 (DOCX)

Table S1 Multivariate analysis of exosomal miRNAs secreted from colon cancer cell lines.
(DOCX)

Table S2 The 69 up-regulated miRNAs (P<0.05, Welch's t-test) in serum exosomes of CRC patients.
(DOCX)

Table S3 The 52 up-regulated miRNAs (P<0.05, Welch's t-test) in five colon cancer cell lines.
(DOCX)

Table S4 Serum levels of 16 miRNAs in CRC patients (n = 29) before and after surgical resection.
(DOCX)

Table S5 Multivariate analysis of the levels of CEA, CA19-9, and eight miRNAs in CRC patients.
(DOCX)

Table S6 Characteristics of the HC and CRC patients used in the qRT-PCR analyses.
(DOCX)
