# Supplementary figures and images for: Circulating Exosomal microRNAs as Biomarkers of Colon Cancer
Source: PLoS One. 2014 Apr 4;9(4):e92921. doi: 10.1371/journal.pone.0092921 (PMC3976275; doi:10.1371/journal.pone.0092921)

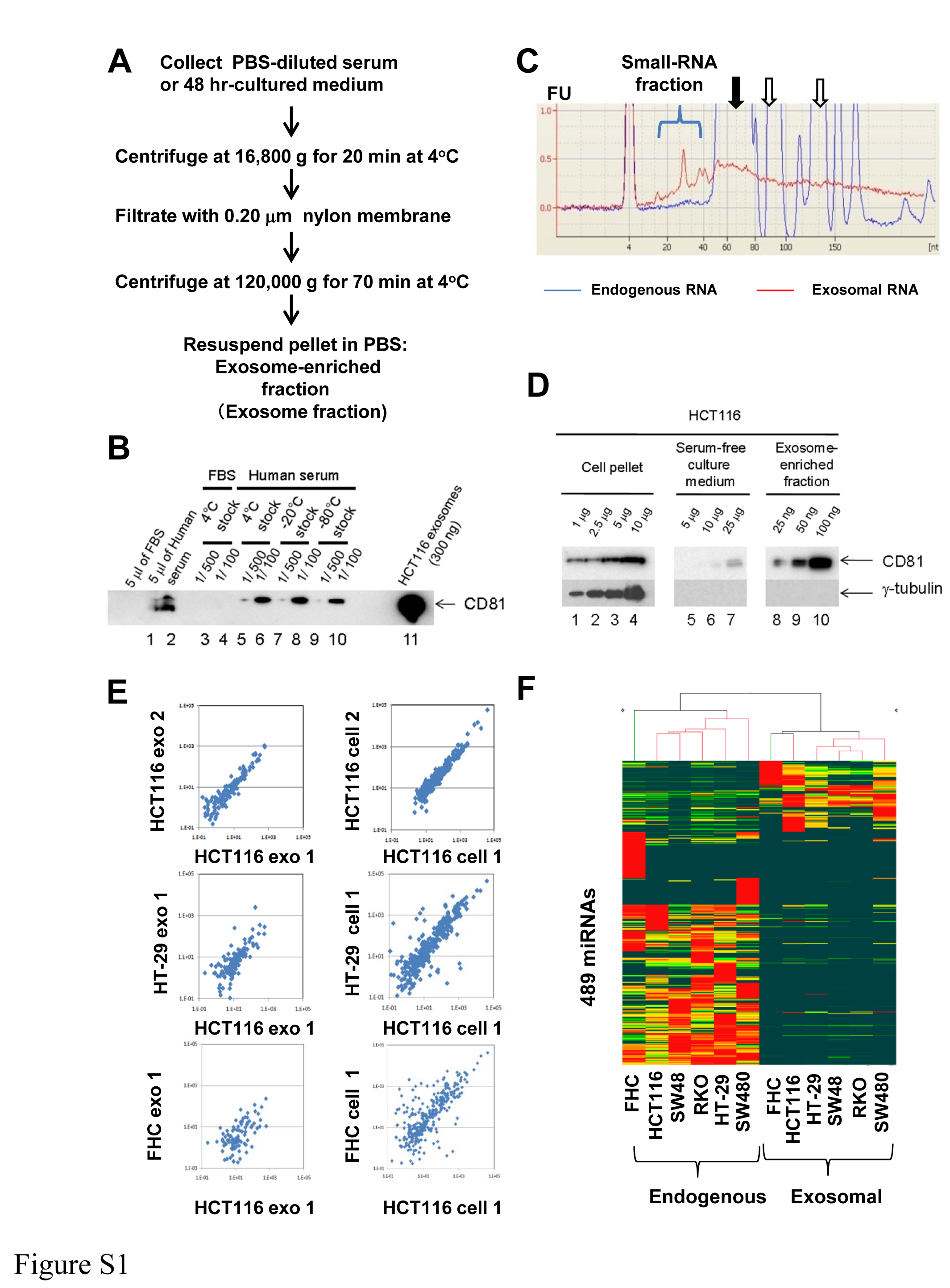

Supplement: Figure S1 — Preparation of exosome-enriched fractions from serum or cell culture medium. (A) Overview of the exosome-enrichment procedure. (B) Immunoblot analysis of CD81 levels in human sera prior to exosome-enrichment and before (lane 2) or after overnsight storage at 4°C (lanes 5 and 6), −20°C (lanes 7 and 8), or −80°C (lanes 9 and 10). Fetal bovine serum (FBS) before (lane 1) and after overnight storage at 4°C (lanes 3 and 4) was used as a control. The exosome-enriched fraction was then prepared from 1 ml of serum using the ultracentrifugation method described in the Materials and Methods section (lane 11). Five microliters of each serum sample, or 300 ng of the enriched fraction was loaded onto a 10–20% gradient SDS-polyacrylamide gel. CD81, an exosome marker, was detected using a specific antibody. (C) Detection of the small RNA fraction of cellular total RNAs (endogenous) and exosomal RNAs (exosomal) from HCT116 cells (30 ng each). The RNAs were loaded onto 5–150 nt small RNA chips (Agilent) and capillary electrophoresed using an Agilent 2100 Bioanalyzer. A small RNA fraction containing miRNAs was detectable at approximately 10–40 nt. The peak at 4 nt represents a size marker. The filled and open arrows indicate 5S tRNA and small rRNAs, respectively. FU, fluorescence units. (D) Immunoblot analysis of CD81 expression in serum-free culture medium of HCT116 cells before and after exosome-enrichment. The specified amounts of protein samples from the cell pellet, culture medium, and exosome-enriched fractions were subjected to immunoblot analysis using antibodies targeting CD81 or anti-γ-tubulin, a representative intracellular protein. Lanes 1–4, cell pellet; lanes 5–7, serum-free culture medium; lanes 8–10, exosome-enriched fraction. (E) Scatter plots of normalized signal intensities (%) of exosomal miRNAs (left panels) and endogenous cellular miRNAs (right panels) in the indicated cell lines. (F) Hierarchical clustering of endogenous and exosomal miRNAs in the normal FHC ce [file pone.0092921.s001.tif]

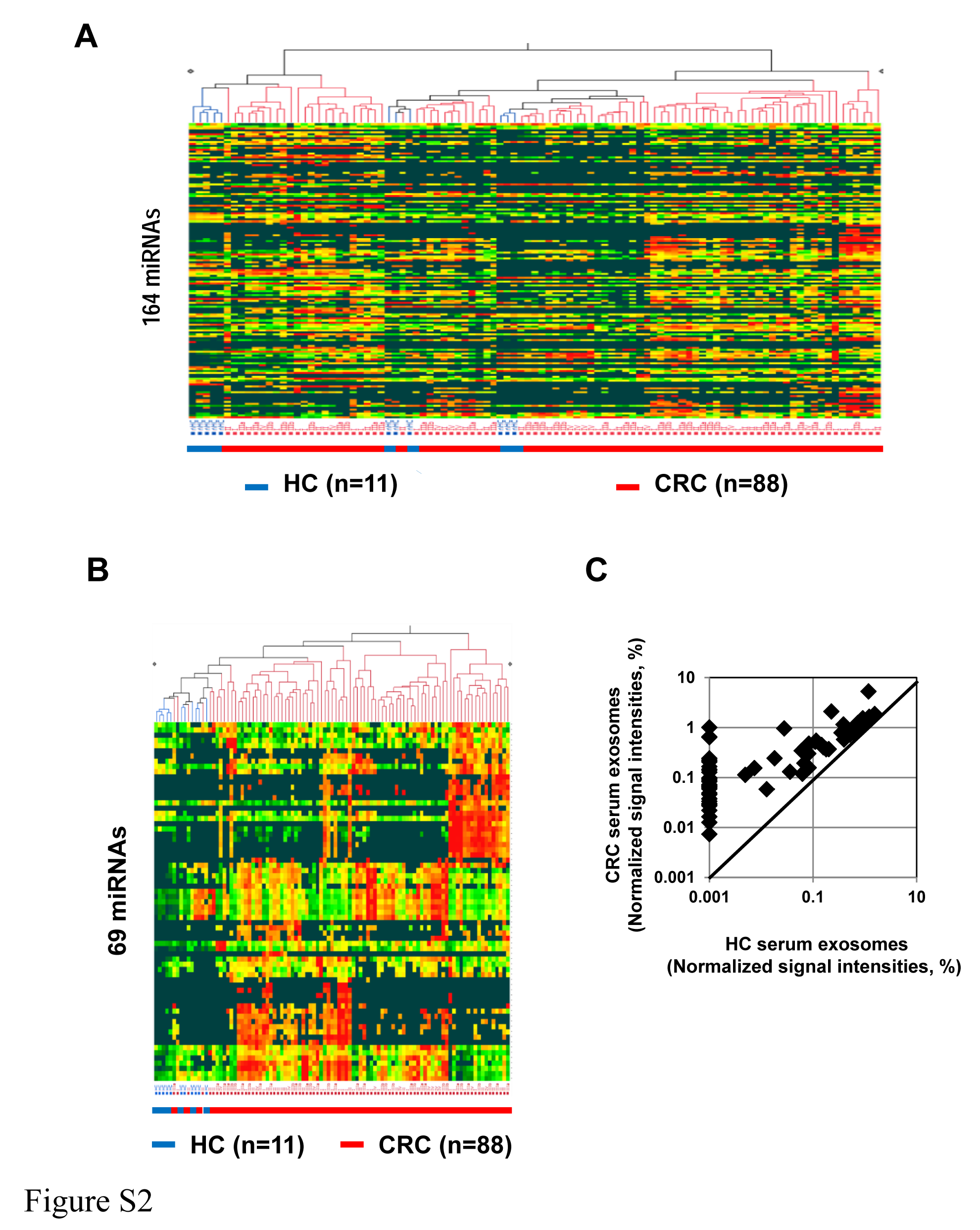

Supplement: Figure S2 — Microarray analysis of miRNA profiles in exosome-enriched serum samples from CRC patients and HCs. (A) Hierarchical clustering of exosomal miRNAs in samples from 11 HCs and 88 CRC patients (TNM: stage I, n = 20; stage II, n = 20; stage IIIa, n = 20; stage IIIb, n = 16; stage IV, n = 12). The blue and red shading indicates the HC and CRC patients, respectively. The signal intensities of each miRNA are shown as a percentage of the total signal intensity on the array. A total of 64 miRNAs were detected in all serum samples examined. (B) Hierarchical clustering of the 69 miRNAs that were expressed at significantly higher levels in CRC patients than HCs (P<0.05 by Welch's t-test). (C) Scatter plot of the normalized signal intensities of the 69 exosomal miRNAs that were up-regulated in CRC patients (y-axis) compared with HCs (x-axis). The data represent the mean normalized signal intensities of each miRNA from the CRC and HC patients. (TIF) [file pone.0092921.s002.tif]

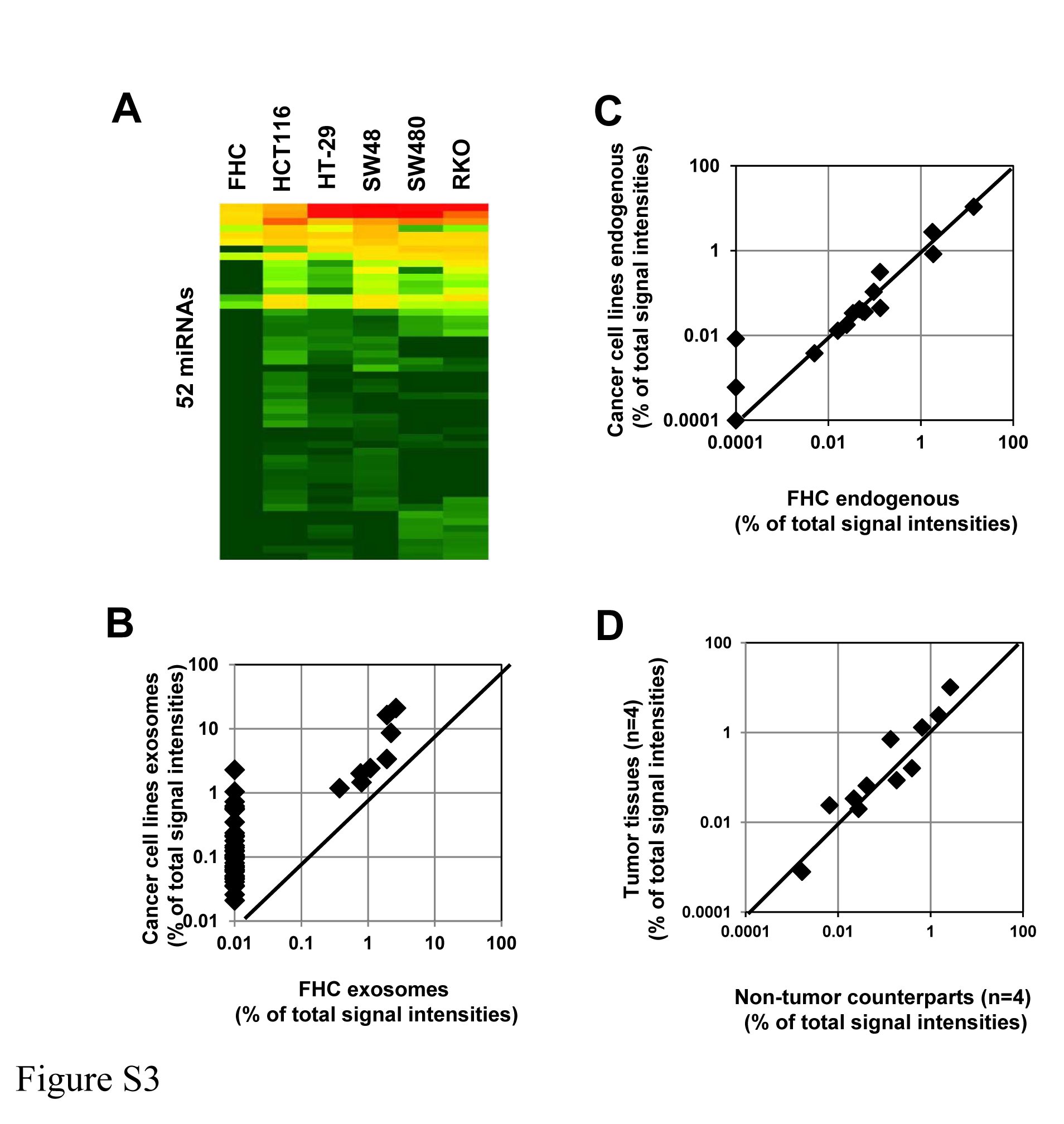

Supplement: Figure S3 — Microarray analysis of exosomal miRNAs from colon cancer cell lines and endogenous expression levels of miRNAs. (A, B) The expression levels of the 52 miRNAs that were secreted from five colon cancer cells at significantly higher levels than from FHC cells (P<0.05). The data represent the mean normalized signal intensities of n = 3 independent microarray experiments. (C) Scatter plot of the normalized signal intensities (%) representing the endogenous expression levels of the 16 miRNAs that were commonly up-regulated in cancer cell lines and serum samples from CRC patients. The endogenous expression levels of these miRNAs were measured in FHC cells and five colon cancer cell lines The data represent the mean normalized signal intensities of n = 2 independent experiments. (D) Scatter plot of the normalized signal intensities (%) representing the endogenous expression levels of the 16 miRNAs that were commonly up-regulated in cancer cell lines and serum samples from CRC patients. The endogenous expression levels of these miRNAs were measured in cancerous lesions (tumor tissues) and matched normal tissue sections from CRC patients. The data represent the mean normalized signal intensities of each miRNA in four different CRC patients. (TIF) [file pone.0092921.s003.tif]

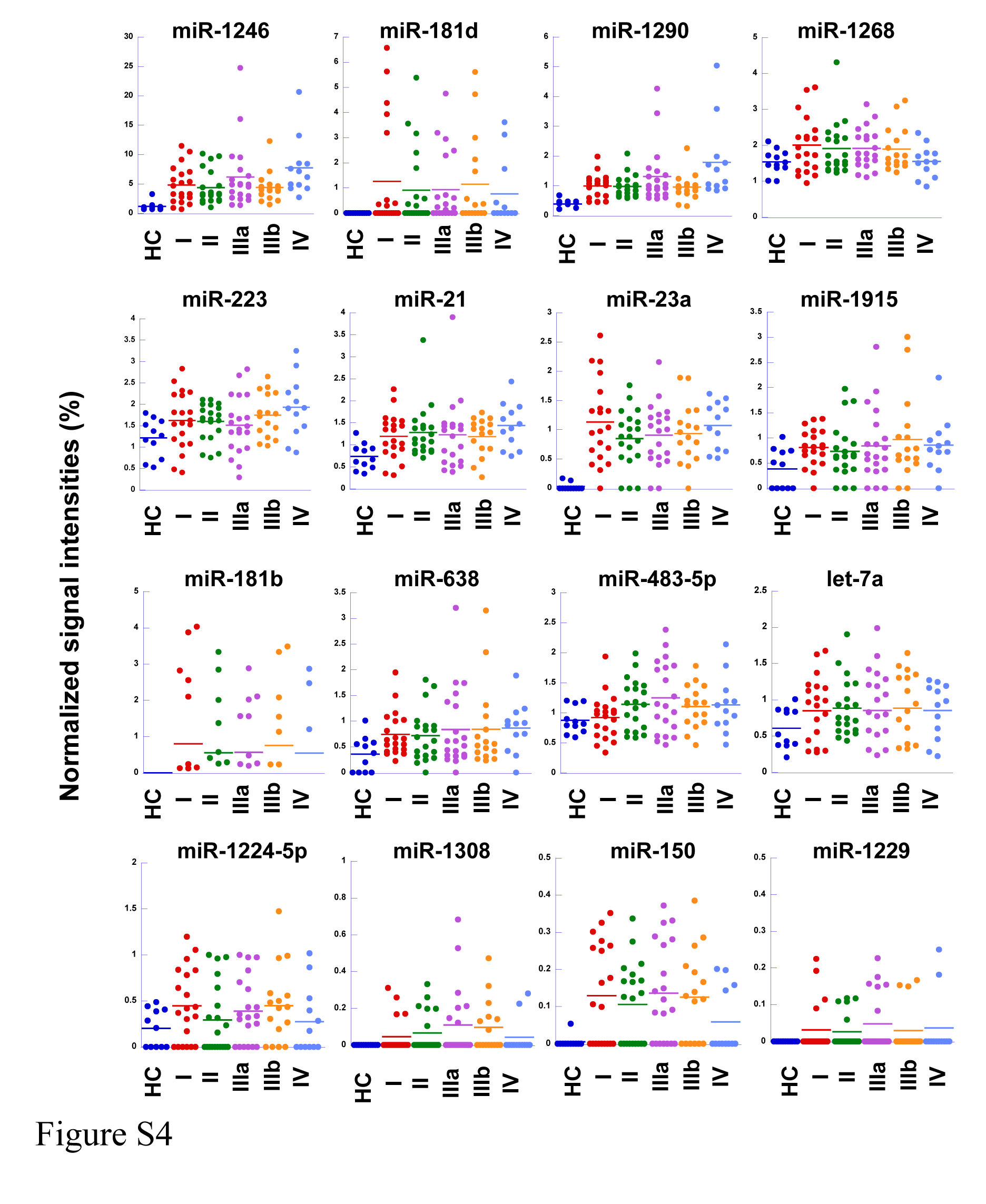

Supplement: Figure S4 — The relationship between the TNM stage and expression levels of the 16 up-regulated miRNAs. Serum exosomal miRNA levels in 11 HCs and 88 CRC patients, classified according to the TNM stage of the disease (stage I, n = 20; stage II, n = 20; stage IIIa, n = 20; stage IIIb, n = 16; and stage IV, n = 12). The signal intensities were normalized to the total signal intensity of the microarray. The horizontal lines indicate the mean normalized signal intensity for each group. (TIF) [file pone.0092921.s004.tif]

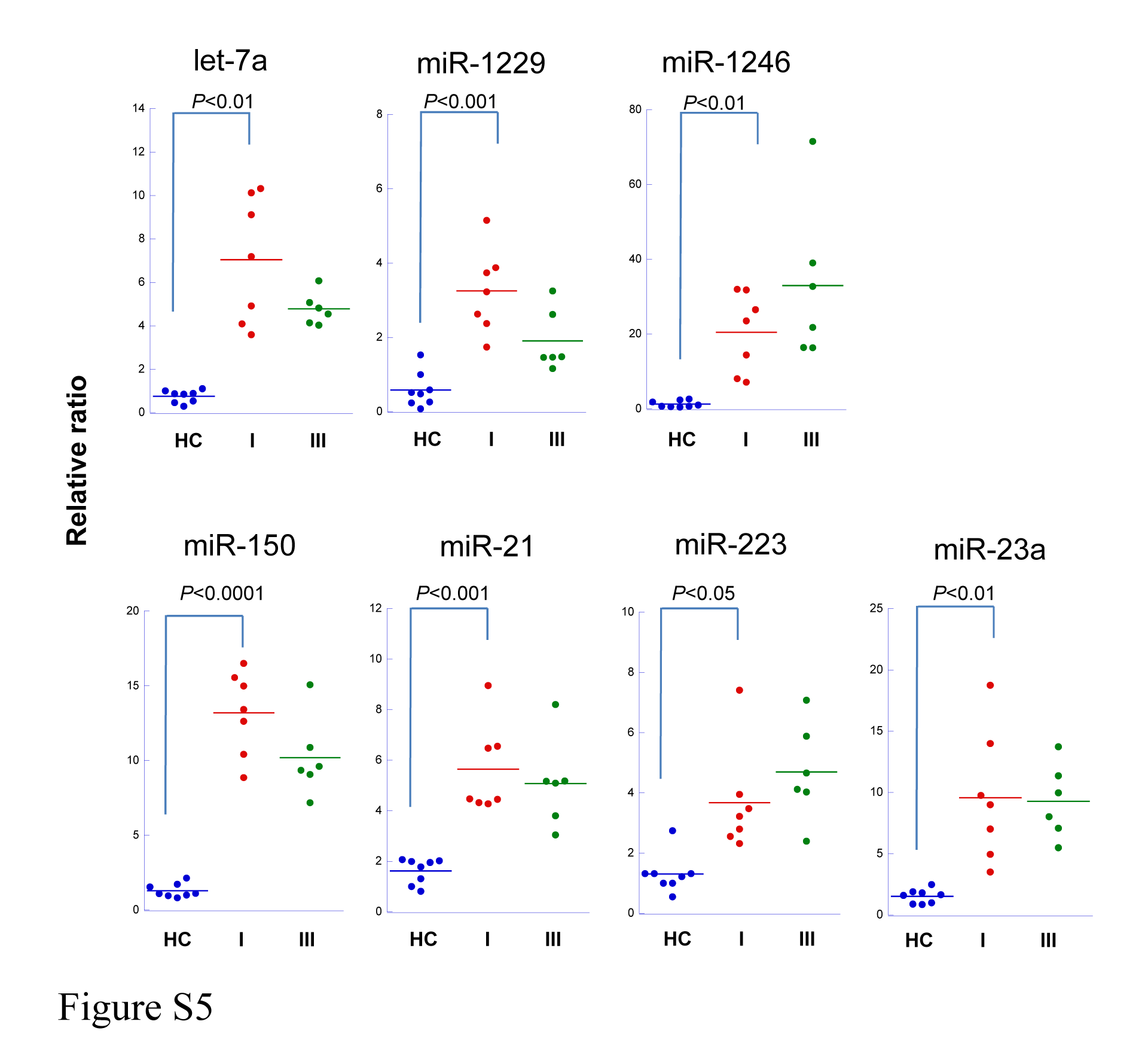

Supplement: Figure S5 — Validation of CRC-associated increases in the expression of seven miRNAs in serum exosomes by qRT-PCR. Expression levels of seven selected miRNAs were validated in an independent set of serum exosomes from HCs (n = 8) and TNM stage I (n = 7) and stage III (n = 6) CRC patients. The comparative Ct method was used to quantify the levels of exosomal miRNAs in HC and CRC patients. The relative ratio was calculated using the 2−ΔΔCt method. The Ct value of miR-451 was used as an internal standard. Each data point was normalized to a representative HC sample. Statistically significant differences were determined by Welch's t-test. Comparisons between the HC and stage III data were also made and all differences were statistically significant (P<0.05) (TIF) [file pone.0092921.s005.tif]
