# Supplementary material for: Circulating Exosomal microRNAs as Biomarkers of Colon Cancer
Source: PLoS One. 2014 Apr 4;9(4):e92921. doi: 10.1371/journal.pone.0092921 (PMC3976275; doi:10.1371/journal.pone.0092921)
Supplement: Method S1 — (DOCX) [file pone.0092921.s006.docx]

**MATERIALS AND METHODS**

**Enrichment of exosomes from frozen blood samples**

Exosome-enriched fractions were prepared from 1 ml samples of human serum that had been stored overnight at 4°C, −20°C or −80°C. A step-wise centrifugation method was used to prepare the fractions (Figure S1A). The pellet was resuspended in 500 µl of PBS and then 1 µl or 5 µl of the sample was mixed with an equal volume of SDS sample buffer and subjected to immunoblot analysis.

**Immunoblot analysis**

Protein concentrations were determined using the CBQCA Protein Quantitation Kit (Invitrogen). Equal amounts of proteins were electrophoresed through a 10–20% SDS-polyacrylamide gradient gel and then transferred onto a PVDF membrane using a standard method. The membrane was incubated overnight at 4°C with the primary antibody diluted in blocking buffer comprising 5% non-fat skimmed milk, 0.1% Tween-20, 50 mM Tris-HCl (pH 8.0), and 150 mM NaCl. The membrane was then incubated with an HRP-conjugated secondary antibody diluted in the same buffer for 60 min at room temperature. The immunoreactive bands were visualized by chemiluminescence using the West Dura Extended Duration Substrate Kit (Thermo Scientific). The antibodies used in the study were as follows: anti-CD81 (1.3.3.22) mouse mAb (Santa Cruz Biotechnology), anti-γ-tubulin mouse pAb (Sigma), and horse-radish peroxidase conjugated anti-mouse IgG (GE Healthcare). The exosomal marker CD81 (TAPA-1) was detected using a specific antibody from Santa Cruz.

**Profiling and identification of exosomal miRNAs from colon cancer cell lines**

The expression levels of exosomal and endogenous miRNAs in colon cancer cell lines were determined. After growth of the cells for 48 h, the culture medium was ultracentrifuged as described in the Materials and Methods section of the main text. The enrichment of CD81 and small RNAs in the exosomal fractions (pellets) was confirmed by immunoblotting and capillary electrophoresis, respectively. The exosomal miRNA profiles in five colon cancer cell lines and the FHC cell line were determined using miRNA microarrays, as described in the Materials and Methods section of the main text. A representative correlation plot was generated to demonstrate reproducible detection of exosomal miRNAs in independent experiments. Calculations of correlation coefficients (r) were also used to detect similarities between the exosomal miRNA profiles of the five different cancer cell lines examined.
