# Supplementary material for: Circulating Exosomal microRNAs as Biomarkers of Colon Cancer
Source: PLoS One. 2014 Apr 4;9(4):e92921. doi: 10.1371/journal.pone.0092921 (PMC3976275; doi:10.1371/journal.pone.0092921)
Supplement: Table S1 — Multivariate analysis of exosomal miRNAs secreted from colon cancer cell lines. (DOCX) [file pone.0092921.s007.docx]

**Table S1.** Multivariate analysis of exosomal miRNAs secreted from colon cancer cell lines.

|  | FHC exo 2 | HCT116 exo 2 | HT29 exo 2 | SW48 exo 2 | SW480 exo 2 | RKO exo 2 |
| --- | --- | --- | --- | --- | --- | --- |
| FHC exo 1 | 0.8767* | 0.6435 | 0.4259 | 0.4529 | 0.4645 | 0.3399 |
|  | (<0.0001) | (<0.0001) | (<0.0001) | (<0.0001) | (<0.0001) | (0.0002) |
| HCT116 exo 1 | 0.5502 | 0.9455 | 0.6791 | 0.7593 | 0.5341 | 0.4235 |
|  | (<0.0001) | (<0.0001) | (<0.0001) | (<0.0001) | (<0.0001) | (<0.0001) |
| HT29 exo 1 | 0.5214 | 0.5473 | 0.9820 | 0.8818 | 0.8421 | 0.7906 |
|  | (<0.0001) | (<0.0001) | (<0.0001) | (<0.0001) | (<0.0001) | (<0.0001) |
| SW48 exo 1 | 0.4441 | 0.7594 | 0.9539 | 0.9541 | 0.6984 | 0.7654 |
|  | (<0.0001) | (<0.0001) | (<0.0001) | (<0.0001) | (<0.0001) | (<0.0001) |
| SW480 exo 1 | 0.4903 | 0.6709 | 0.8687 | 0.8509 | 0.9308 | 0.8558 |
|  | (<0.0001) | (<0.0001) | (<0.0001) | (<0.0001) | (<0.0001) | (<0.0001) |
| RKO exo 1 | 0.4518 | 0.4445 | 0.8306 | 0.7504 | 0.8711 | 0.9885 |
|  | (<0.0001) | (<0.0001) | (<0.0001) | (<0.0001) | (<0.0001) | (<0.0001) |
| *Correlation coefficients (r) are shown. The numbers in parentheses indicate the *P* values. The exo 1 and exo 2 samples were enriched in independent experiments. | | | | | | |
