# Supplementary material for: Circulating Exosomal microRNAs as Biomarkers of Colon Cancer
Source: PLoS One. 2014 Apr 4;9(4):e92921. doi: 10.1371/journal.pone.0092921 (PMC3976275; doi:10.1371/journal.pone.0092921)
Supplement: Table S2 — The 69 up-regulated miRNAs (P<0.05, Welch's t-test) in serum exosomes of CRC patients. (DOCX) [file pone.0092921.s008.docx]

**Table S2.** The 69 up-regulated miRNAs (*P* < 0.05, Welch’s t-test) in serum exosomes of CRC patients.

|  | Stage I | Stage II | Stage IIIa | Stage IIIb | Stage IV | HC | CRC | Fold Change |
| --- | --- | --- | --- | --- | --- | --- | --- | --- |
| Up-regulated miRNAs | 53 | 49 | 46 | 45 | 30 | mean*‡ | mean*‡ | CRC/HC |
| hsa-let-7a |  | ○ |  |  |  | 0.61 | 0.87 | 1.4 |
| hsa-let-7b | ○ | ○ | ○ | ○ | ○ | 0.11 | 0.53 | 4.8 |
| hsa-let-7b# | ○ | ○ | ○ | ○ | ○ | 0.01 | 0.05 | 5.0 |
| hsa-let-7c | ○ | ○ | ○ | ○ |  | 0.01 | 0.07 | 6.8 |
| hsa-let-7f |  | ○ |  |  |  | 0.60 | 0.80 | 1.3 |
| hsa-let-7f-1# | ○ | ○ | ○ | ○ |  | 0.01 | 0.04 | 3.8 |
| hsa-miR-107 | ○ | ○ | ○ | ○ | ○ | 0.02 | 0.25 | 13.7 |
| hsa-miR-10a | ○ |  |  |  |  | 0.01 | 0.01 | 1.3 |
| hsa-miR-10b | ○ | ○ | ○ | ○ | ○ | 0.01 | 0.10 | 9.9 |
| hsa-miR-1180 | ○ | ○ | ○ | ○ |  | 0.01 | 0.13 | 12.9 |
| hsa-miR-1207-5p | ○ | ○ | ○ | ○ |  | 0.86 | 1.30 | 1.5 |
| hsa-miR-1224-5p | ○ |  |  |  |  | 0.20 | 0.37 | 1.9 |
| hsa-miR-1225-3p |  |  |  |  | ○ | 0.39 | 0.58 | 1.5 |
| hsa-miR-1228 |  |  |  | ○ | ○ | 0.83 | 1.19 | 1.4 |
| hsa-miR-1229 |  | ○ | ○ |  |  | 0.01 | 0.03 | 3.4 |
| hsa-miR-1234 |  |  |  |  | ○ | 0.73 | 1.00 | 1.4 |
| hsa-miR-1237 | ○ | ○ | ○ | ○ | ○ | 0.01 | 0.23 | 22.5 |
| hsa-miR-1238 |  |  |  |  | ○ | 0.63 | 0.84 | 1.3 |
| hsa-miR-124 | ○ |  |  | ○ |  | 0.06 | 0.12 | 1.9 |
| hsa-miR-1246 | ○ | ○ | ○ | ○ | ○ | 1.17 | 5.36 | 4.6 |
| hsa-miR-1249 | ○ | ○ |  | ○ |  | 0.01 | 0.06 | 6.0 |
| hsa-miR-126 | ○ | ○ | ○ | ○ | ○ | 0.23 | 2.13 | 9.4 |
| hsa-miR-126* | ○ | ○ | ○ | ○ |  | 0.01 | 0.09 | 8.7 |
| hsa-miR-1268 | ○ |  | ○ |  |  | 1.54 | 1.88 | 1.2 |
| hsa-miR-1280 |  |  |  |  | ○ | 0.51 | 0.73 | 1.4 |
| hsa-miR-1281 | ○ | ○ | ○ | ○ | ○ | 0.08 | 0.48 | 5.8 |
| hsa-miR-129# | ○ | ○ | ○ | ○ |  | 0.01 | 0.07 | 6.5 |
| hsa-miR-1290 | ○ | ○ | ○ | ○ | ○ | 0.39 | 1.16 | 3.0 |
| hsa-miR-129-3p | ○ | ○ | ○ | ○ |  | 0.01 | 0.07 | 6.9 |
| hsa-miR-1306 | ○ | ○ | ○ | ○ |  | 0.01 | 0.21 | 20.8 |
| hsa-miR-1307 | ○ |  |  |  |  | 0.01 | 0.07 | 6.6 |
| hsa-miR-1308 |  | ○ | ○ | ○ |  | 0.01 | 0.07 | 7.3 |
| hsa-miR-150 | ○ | ○ | ○ | ○ |  | 0.01 | 0.12 | 23.0 |
| hsa-miR-15a | ○ | ○ | ○ | ○ | ○ | 0.16 | 0.41 | 2.6 |
| hsa-miR-181b | ○ | ○ | ○ | ○ |  | 0.01 | 0.65 | 64.9 |
| hsa-miR-181d | ○ | ○ | ○ | ○ |  | 0.01 | 1.02 | 101.8 |
| hsa-miR-1825 | ○ | ○ | ○ | ○ | ○ | 0.12 | 0.55 | 4.8 |
| hsa-miR-1915 | ○ |  | ○ | ○ | ○ | 0.39 | 0.83 | 2.2 |
| hsa-miR-21 | ○ | ○ | ○ | ○ | ○ | 0.73 | 1.25 | 1.7 |
| hsa-miR-22 | ○ | ○ | ○ | ○ | ○ | 0.06 | 0.35 | 5.8 |
| hsa-miR-223 |  | ○ |  | ○ | ○ | 1.22 | 1.66 | 1.4 |
| hsa-miR-23a | ○ | ○ | ○ | ○ | ○ | 0.03 | 0.97 | 34.8 |
| hsa-miR-23b | ○ | ○ | ○ | ○ | ○ | 0.01 | 0.08 | 8.4 |
| hsa-miR-24 | ○ | ○ | ○ |  |  | 0.01 | 0.09 | 9.4 |
| hsa-miR-26a | ○ | ○ | ○ | ○ | ○ | 0.01 | 0.17 | 16.8 |
| hsa-miR-26b | ○ | ○ | ○ | ○ | ○ | 0.15 | 0.45 | 3.1 |
| hsa-miR-27a | ○ | ○ | ○ | ○ |  | 0.01 | 0.16 | 22.4 |
| hsa-miR-27b | ○ |  |  |  |  | 0.01 | 0.03 | 2.9 |
| hsa-miR-296-5p |  | ○ | ○ |  |  | 0.01 | 0.04 | 3.8 |
| hsa-miR-29a | ○ |  |  |  |  | 0.01 | 0.02 | 2.2 |
| hsa-miR-30b | ○ |  |  |  |  | 0.01 | 0.02 | 1.7 |
| hsa-miR-30d | ○ |  |  |  |  | 0.01 | 0.05 | 4.6 |
| hsa-miR-365 |  | ○ |  |  |  | 0.01 | 0.03 | 3.0 |
| hsa-miR-432 | ○ | ○ | ○ | ○ |  | 0.01 | 0.25 | 24.5 |
| hsa-miR-483-5p |  | ○ | ○ | ○ |  | 0.88 | 1.11 | 1.3 |
| hsa-miR-526b | ○ | ○ | ○ |  |  | 0.04 | 0.13 | 3.7 |
| hsa-miR-548c-3p | ○ | ○ | ○ | ○ | ○ | 0.01 | 0.14 | 14.3 |
| hsa-miR-548f |  | ○ |  |  |  | 0.01 | 0.03 | 2.8 |
| hsa-miR-574-5p |  | ○ | ○ | ○ |  | 0.18 | 0.37 | 2.1 |
| hsa-miR-584 | ○ |  |  | ○ |  | 0.72 | 1.14 | 1.6 |
| hsa-miR-595 | ○ | ○ | ○ | ○ |  | 0.01 | 0.15 | 14.8 |
| hsa-miR-623 | ○ |  |  |  |  | 0.08 | 0.31 | 3.8 |
| hsa-miR-634 | ○ |  | ○ |  |  | 0.01 | 0.06 | 4.5 |
| hsa-miR-638 | ○ | ○ | ○ | ○ | ○ | 0.35 | 0.79 | 2.2 |
| hsa-miR-654-5p | ○ | ○ | ○ | ○ | ○ | 0.01 | 0.66 | 66.2 |
| hsa-miR-766 | ○ |  |  |  | ○ | 0.08 | 0.16 | 1.9 |
| hsa-miR-92b |  |  |  | ○ |  | 0.01 | 0.02 | 1.7 |
| hsa-miR-933 | ○ | ○ | ○ | ○ | ○ | 0.07 | 0.20 | 2.9 |
| hsa-miR-940 | ○ | ○ | ○ | ○ | ○ | 0.91 | 1.54 | 1.7 |
| * The signal intensities of the miRNAs are shown as percentages of the total signal intensity. | | | | | | | | |
| ‡ Normalized intensities of undetectable miRNAs in serum exosomes are listed as 0.01%. | | | | | | | | |
| # Star-form of the specified miRNA. | | | | | | | | |
