# Supplementary material for: Circulating Exosomal microRNAs as Biomarkers of Colon Cancer
Source: PLoS One. 2014 Apr 4;9(4):e92921. doi: 10.1371/journal.pone.0092921 (PMC3976275; doi:10.1371/journal.pone.0092921)
Supplement: Table S3 — The 52 up-regulated miRNAs (P<0.05, Welch's t-test) in five colon cancer cell lines. (DOCX) [file pone.0092921.s009.docx]

**Table S3.** The 52 up-regulated miRNAs (*P* < 0.05, Welch’s t-test) in five colon cancer cell lines.

| miRNA | FHC | | HCT 116 | | HT29 | | SW48 | | SW480 | | RKO | | Cancers | Fold Change | *P* value |
| --- | --- | --- | --- | --- | --- | --- | --- | --- | --- | --- | --- | --- | --- | --- | --- |
| (n=3) | mean*† | SD*† | mean*† | SD*† | mean*† | SD*† | mean*† | SD*† | mean*† | SD*† | mean*† | SD*† | mean*† | (/FHC) |  |
| hsa-let-7a | 0.01 | 0.00 | 0.01 | 0.00 | 0.10 | 0.10 | 0.01 | 0.00 | 0.14 | 0.12 | 0.23 | 0.04 | 0.09 | 9.4 | 0.008 |
| hsa-miR-1181 | 0.01 | 0.00 | 0.01 | 0.00 | 0.01 | 0.00 | 0.38 | 0.31 | 0.14 | 0.10 | 0.11 | 0.08 | 0.13 | 12.5 | 0.039 |
| hsa-miR-1183 | 0.01 | 0.00 | 0.20 | 0.15 | 0.07 | 0.05 | 0.14 | 0.12 | 0.11 | 0.08 | 0.22 | 0.06 | 0.15 | 15.0 | 0.000 |
| hsa-miR-1202 | 0.52 | 0.15 | 1.76 | 0.73 | 0.73 | 0.20 | 1.24 | 0.36 | 0.77 | 0.11 | 0.73 | 0.09 | 1.05 | 2.0 | 0.015 |
| hsa-miR-1224-5p | 0.01 | 0.00 | 0.35 | 0.02 | 0.08 | 0.06 | 0.18 | 0.05 | 0.22 | 0.08 | 0.07 | 0.05 | 0.18 | 17.9 | 0.000 |
| hsa-miR-1225-5p | 0.80 | 0.25 | 1.19 | 0.38 | 0.66 | 0.07 | 1.52 | 0.33 | 1.60 | 0.24 | 2.33 | 0.37 | 1.46 | 1.8 | 0.031 |
| hsa-miR-1229 | 0.01 | 0.00 | 0.12 | 0.05 | 0.02 | 0.02 | 0.08 | 0.08 | 0.01 | 0.00 | 0.01 | 0.00 | 0.05 | 4.5 | 0.020 |
| hsa-miR-1246 | 1.08 | 0.48 | 0.54 | 0.17 | 0.41 | 0.17 | 0.60 | 0.35 | 0.33 | 0.11 | 0.26 | 0.07 | 2.43 | 2.2 | 0.048 |
| hsa-miR-125a-3p | 0.01 | 0.53 | 2.94 | 1.14 | 1.28 | 0.83 | 3.70 | 2.94 | 2.04 | 1.19 | 2.20 | 0.54 | 0.05 | 5.0 | 0.022 |
| hsa-miR-125b | 0.01 | 0.00 | 0.08 | 0.06 | 0.06 | 0.05 | 0.11 | 0.11 | 0.01 | 0.00 | 0.01 | 0.00 | 0.04 | 3.5 | 0.049 |
| hsa-miR-1268 | 1.91 | 0.00 | 0.08 | 0.07 | 0.01 | 0.00 | 0.10 | 0.07 | 0.01 | 0.00 | 0.01 | 0.00 | 3.37 | 1.8 | 0.011 |
| hsa-miR-127-3p | 0.01 | 0.23 | 4.02 | 1.41 | 3.19 | 1.63 | 4.84 | 2.27 | 2.24 | 0.98 | 2.57 | 0.83 | 0.06 | 5.9 | 0.017 |
| hsa-miR-1274a | 0.01 | 0.00 | 0.12 | 0.09 | 0.07 | 0.06 | 0.11 | 0.09 | 0.01 | 0.00 | 0.01 | 0.00 | 0.06 | 6.3 | 0.048 |
| hsa-miR-1275 | 0.01 | 0.00 | 0.48 | 0.15 | 0.16 | 0.02 | 0.75 | 0.11 | 0.48 | 0.15 | 0.90 | 0.25 | 0.55 | 55.3 | 0.000 |
| hsa-miR-1290 | 0.01 | 0.00 | 0.66 | 0.29 | 0.38 | 0.26 | 0.81 | 0.64 | 0.44 | 0.35 | 0.58 | 0.11 | 0.57 | 57.3 | 0.000 |
| hsa-miR-1308 | 0.01 | 0.00 | 0.29 | 0.04 | 0.13 | 0.03 | 0.07 | 0.05 | 0.01 | 0.00 | 0.01 | 0.00 | 0.10 | 9.8 | 0.006 |
| hsa-miR-134 | 0.01 | 0.00 | 0.13 | 0.11 | 0.00 | 0.00 | 0.16 | 0.15 | 0.01 | 0.00 | 0.24 | 0.05 | 0.11 | 10.6 | 0.007 |
| hsa-miR-150 | 0.01 | 0.00 | 0.03 | 0.02 | 0.07 | 0.05 | 0.01 | 0.00 | 0.05 | 0.05 | 0.07 | 0.05 | 0.05 | 4.6 | 0.003 |
| hsa-miR-150# | 0.01 | 0.00 | 0.15 | 0.12 | 0.04 | 0.03 | 0.01 | 0.00 | 0.08 | 0.06 | 0.09 | 0.02 | 0.07 | 7.3 | 0.003 |
| hsa-miR-15b | 0.01 | 0.00 | 0.01 | 0.00 | 0.01 | 0.00 | 0.01 | 0.00 | 0.23 | 0.21 | 0.29 | 0.05 | 0.11 | 10.5 | 0.030 |
| hsa-miR-16 | 0.01 | 0.00 | 0.01 | 0.00 | 0.06 | 0.05 | 0.01 | 0.00 | 0.23 | 0.22 | 0.21 | 0.03 | 0.10 | 9.9 | 0.020 |
| hsa-miR-181b | 0.01 | 0.00 | 0.20 | 0.02 | 0.11 | 0.03 | 0.10 | 0.09 | 0.01 | 0.00 | 0.01 | 0.00 | 0.08 | 8.1 | 0.004 |
| hsa-miR-181d | 0.01 | 0.00 | 0.21 | 0.05 | 0.01 | 0.00 | 0.10 | 0.10 | 0.01 | 0.00 | 0.01 | 0.00 | 0.06 | 6.2 | 0.033 |
| hsa-miR-1826 | 0.01 | 0.00 | 0.07 | 0.05 | 0.07 | 0.02 | 0.12 | 0.08 | 0.01 | 0.00 | 0.01 | 0.00 | 0.05 | 5.1 | 0.009 |
| hsa-miR-188-5p | 0.01 | 0.00 | 0.34 | 0.07 | 0.11 | 0.09 | 0.25 | 0.05 | 0.01 | 0.00 | 0.01 | 0.00 | 0.14 | 14.0 | 0.003 |
| hsa-miR-1915 | 1.92 | 1.91 | 6.99 | 2.79 | 26.81 | 5.41 | 19.01 | 4.50 | 17.41 | 6.34 | 11.63 | 1.09 | 16.37 | 8.5 | 0.000 |
| hsa-miR-195# | 0.01 | 0.00 | 0.17 | 0.01 | 0.01 | 0.00 | 0.06 | 0.07 | 0.01 | 0.00 | 0.01 | 0.00 | 0.05 | 4.7 | 0.033 |
| hsa-miR-21 | 0.01 | 0.00 | 0.01 | 0.00 | 0.09 | 0.10 | 0.01 | 0.00 | 0.24 | 0.23 | 0.15 | 0.01 | 0.10 | 9.8 | 0.025 |
| hsa-miR-223 | 0.01 | 0.00 | 0.16 | 0.06 | 0.18 | 0.11 | 0.11 | 0.08 | 0.28 | 0.23 | 0.45 | 0.06 | 0.24 | 23.7 | 0.000 |
| hsa-miR-23a | 0.01 | 0.00 | 0.06 | 0.05 | 0.06 | 0.05 | 0.01 | 0.00 | 0.17 | 0.13 | 0.21 | 0.01 | 0.10 | 10.0 | 0.003 |
| hsa-miR-25 | 0.01 | 0.00 | 0.15 | 0.07 | 0.15 | 0.08 | 0.09 | 0.07 | 0.29 | 0.21 | 0.37 | 0.02 | 0.21 | 20.8 | 0.000 |
| hsa-miR-297 | 0.01 | 0.00 | 0.29 | 0.06 | 0.07 | 0.07 | 0.01 | 0.00 | 0.01 | 0.00 | 0.01 | 0.00 | 0.07 | 7.2 | 0.041 |
| hsa-miR-30c-1# | 0.01 | 0.00 | 0.03 | 0.02 | 0.01 | 0.00 | 0.14 | 0.06 | 0.01 | 0.00 | 0.01 | 0.00 | 0.04 | 3.6 | 0.049 |
| hsa-miR-320c | 0.01 | 0.00 | 0.65 | 0.15 | 0.27 | 0.07 | 0.75 | 0.21 | 0.83 | 0.29 | 1.12 | 0.36 | 0.73 | 72.6 | 0.000 |
| hsa-miR-320d | 0.01 | 0.00 | 0.01 | 0.00 | 0.01 | 0.00 | 0.05 | 0.04 | 0.08 | 0.06 | 0.01 | 0.00 | 0.03 | 2.6 | 0.050 |
| hsa-miR-324-3p | 0.01 | 0.00 | 0.25 | 0.02 | 0.20 | 0.02 | 0.17 | 0.15 | 0.01 | 0.00 | 0.01 | 0.00 | 0.12 | 12.3 | 0.002 |
| hsa-miR-338-5p | 0.01 | 0.00 | 0.25 | 0.03 | 0.06 | 0.06 | 0.01 | 0.00 | 0.01 | 0.00 | 0.01 | 0.00 | 0.06 | 6.3 | 0.037 |
| hsa-miR-33b# | 0.01 | 0.00 | 0.00 | 0.00 | 0.01 | 0.00 | 0.01 | 0.00 | 0.30 | 0.07 | 0.24 | 0.08 | 0.11 | 10.8 | 0.013 |
| hsa-miR-345 | 0.01 | 0.00 | 0.00 | 0.00 | 0.01 | 0.00 | 0.01 | 0.00 | 0.15 | 0.13 | 0.20 | 0.12 | 0.07 | 6.9 | 0.044 |
| hsa-miR-34a | 0.01 | 0.00 | 0.26 | 0.04 | 0.16 | 0.07 | 0.22 | 0.11 | 0.01 | 0.00 | 0.01 | 0.00 | 0.13 | 12.7 | 0.002 |
| hsa-miR-371-5p | 0.01 | 0.00 | 0.15 | 0.14 | 0.05 | 0.04 | 0.11 | 0.08 | 0.01 | 0.00 | 0.01 | 0.00 | 0.06 | 6.4 | 0.026 |
| hsa-miR-432 | 0.01 | 0.00 | 0.18 | 0.03 | 0.03 | 0.04 | 0.01 | 0.00 | 0.01 | 0.00 | 0.01 | 0.00 | 0.04 | 4.1 | 0.050 |
| hsa-miR-483-5p | 0.77 | 0.14 | 3.36 | 1.72 | 0.95 | 0.40 | 4.81 | 1.31 | 0.37 | 0.15 | 0.57 | 0.08 | 2.01 | 2.6 | 0.039 |
| hsa-miR-494 | 0.01 | 0.00 | 0.55 | 0.25 | 0.35 | 0.05 | 0.74 | 0.33 | 0.54 | 0.25 | 0.72 | 0.06 | 0.58 | 58.1 | 0.000 |
| hsa-miR-513a-5p | 0.01 | 0.00 | 0.56 | 0.36 | 0.40 | 0.15 | 1.07 | 0.79 | 0.17 | 0.01 | 0.90 | 0.26 | 0.62 | 62.4 | 0.001 |
| hsa-miR-513b | 0.01 | 0.00 | 0.01 | 0.00 | 0.00 | 0.00 | 0.05 | 0.04 | 0.01 | 0.00 | 0.05 | 0.03 | 0.02 | 2.1 | 0.043 |
| hsa-miR-572 | 0.01 | 0.00 | 0.44 | 0.18 | 2.64 | 0.60 | 1.64 | 0.44 | 3.46 | 1.14 | 3.27 | 0.58 | 2.29 | 228.8 | 0.000 |
| hsa-miR-575 | 0.37 | 0.28 | 1.44 | 0.31 | 0.73 | 0.22 | 1.23 | 0.85 | 0.90 | 0.22 | 1.62 | 0.41 | 1.18 | 3.2 | 0.026 |
| hsa-miR-630 | 2.20 | 1.67 | 12.01 | 1.65 | 4.52 | 0.49 | 7.41 | 2.68 | 10.57 | 2.46 | 8.51 | 2.21 | 8.61 | 3.9 | 0.009 |
| hsa-miR-638 | 2.63 | 2.85 | 6.09 | 2.08 | 26.18 | 6.23 | 18.30 | 5.79 | 25.89 | 9.10 | 28.42 | 3.86 | 20.98 | 8.0 | 0.000 |
| hsa-miR-671-5p | 0.01 | 0.00 | 0.31 | 0.13 | 0.16 | 0.03 | 0.25 | 0.02 | 0.48 | 0.15 | 0.57 | 0.13 | 0.35 | 35.1 | 0.000 |
| hsa-miR-92a | 0.01 | 0.00 | 0.18 | 0.05 | 0.15 | 0.08 | 0.07 | 0.05 | 0.29 | 0.23 | 0.40 | 0.04 | 0.22 | 21.7 | 0.000 |
| * The signal intensities of the miRNAs are shown as percentages of the total signal intensity. | | | | | | | | | | | | | | | |
| † Normalized intensities of undetectable miRNAs in exosomes are listed as 0.01%. | | | | | | | | | | | | | | | |
| # Star-form of the specified miRNA. | | | | | | | | | | | | | | | |
