# Supplementary material for: Circulating Exosomal microRNAs as Biomarkers of Colon Cancer
Source: PLoS One. 2014 Apr 4;9(4):e92921. doi: 10.1371/journal.pone.0092921 (PMC3976275; doi:10.1371/journal.pone.0092921)
Supplement: Table S4 — Serum levels of 16 miRNAs in CRC patients (n = 29) before and after surgical resection. (DOCX) [file pone.0092921.s010.docx]

**Table S4.** Serum levels of 16 miRNAs in CRC patients (n = 29) before and after surgical resection.

| miRNA | Pre (before) | | Post (after) | |  |  |
| --- | --- | --- | --- | --- | --- | --- |
|  | mean*† | SD*† |  | mean*† SD*† |  | *P* value‡ |
| hsa-let-7a | 0.73 | 0.39 |  | 0.23 0.35 |  | 0.0000 |
| hsa-miR-1224-5p | 0.60 | 0.36 |  | 0.19 0.25 |  | 0.0001 |
| hsa-miR-1229 | 0.04 | 0.07 |  | 0.01 0.00 |  | 0.0054 |
| hsa-miR-1246 | 5.71 | 3.30 |  | 4.29 2.24 |  | 0.0310 |
| hsa-miR-1268 | 2.11 | 0.85 |  | 3.47 1.23 |  | 0.0000 |
| hsa-miR-1290 | 1.13 | 0.59 |  | 0.98 0.43 |  | 0.3091 |
| hsa-miR-1308 | 0.12 | 0.11 |  | 0.06 0.23 |  | 0.2405 |
| hsa-miR-150 | 0.14 | 0.11 |  | 0.02 0.06 |  | 0.0000 |
| hsa-miR-181b | 1.40 | 1.38 |  | 1.38 1.08 |  | 0.9436 |
| hsa-miR-181d | 2.08 | 2.05 |  | 1.85 1.42 |  | 0.6095 |
| hsa-miR-1915 | 1.03 | 0.54 |  | 2.27 1.15 |  | 0.0000 |
| hsa-miR-21 | 1.04 | 0.50 |  | 0.18 0.27 |  | 0.0000 |
| hsa-miR-223 | 1.42 | 0.57 |  | 0.58 0.44 |  | 0.0000 |
| hsa-miR-23a | 0.97 | 0.44 |  | 0.30 0.55 |  | 0.0000 |
| hsa-miR-483-5p | 0.99 | 0.39 |  | 1.99 1.04 |  | 0.0000 |
| hsa-miR-638 | 0.84 | 0.52 |  | 2.93 1.20 |  | 0.0000 |
| * The signal intensities of the miRNAs are shown as percentages of the total signal intensity. | | | | | | |
| † Normalized intensities of undetectable miRNAs in serum exosomes are listed as 0.01%. | | | | | | |
| ‡ Determined by a Student's *t* -test. | | | | | | |
