# Supplementary material for: Circulating Exosomal microRNAs as Biomarkers of Colon Cancer
Source: PLoS One. 2014 Apr 4;9(4):e92921. doi: 10.1371/journal.pone.0092921 (PMC3976275; doi:10.1371/journal.pone.0092921)
Supplement: Table S5 — Multivariate analysis of the levels of CEA, CA19-9, and eight miRNAs in CRC patients. (DOCX) [file pone.0092921.s011.docx]

**Table S5.** Multivariate analysis of the levels of CEA, CA19-9, and eight miRNAs in CRC patients.

| Correlation coefficient (r) | CEA | CA19-9 |
| --- | --- | --- |
| CEA | 1.000 | 0.075 |
| CA19-9 | 0.075 | 1.000 |
| hsa-let-7a | −0.099 | 0.147 |
| hsa-miR-1224-5p | 0.105 | −0.133 |
| hsa-miR-1229 | −0.092 | −0.066 |
| hsa-miR-1246 | 0.388 | 0.006 |
| hsa-miR-150 | −0.105 | 0.169 |
| hsa-miR-21 | −0.108 | 0.105 |
| hsa-miR-223 | −0.181 | 0.287 |
| hsa-miR-23a | −0.089 | 0.221 |
