# Supplementary material for: Circulating Exosomal microRNAs as Biomarkers of Colon Cancer
Source: PLoS One. 2014 Apr 4;9(4):e92921. doi: 10.1371/journal.pone.0092921 (PMC3976275; doi:10.1371/journal.pone.0092921)
Supplement: Table S6 — Characteristics of the HC and CRC patients used in the qRT-PCR analyses. (DOCX) [file pone.0092921.s012.docx]

**Table S6**. Characteristics of the HC and CRC patients used in the qRT-PCR analyses.

|  | Healthy controls | Colorectal cancer patients | |
| --- | --- | --- | --- |
| TNM stage |  | I | III |
|  | (n = 8) | (n = 7) | (n = 6) |
| Age in years, mean (SD) | 49.7 (10.1) | 62.4 (6.8) | 58.3 (7.6) |
| Range | 38–64 | 54–68 | 46–66 |
| Sex, n (%) |  |  |  |
| Female | 5 (50.0) | 2 (28.6) | 2 (33.3) |
| Male | 5 (50.0) | 5 (71.4) | 4 (66.7) |
| CA19-9 value (U/ml), mean (SD) | – | 10.0 (5.5) | 23.8 (24.5) |
| Range | – | 3–19 | 3–73 |
